# Efficacy and safety of ureteroscopy in children with lower pole renal stones : a machine learning predictive model from the EAU section of endourology

**DOI:** 10.1007/s00345-025-06095-1

**Published:** 2025-11-20

**Authors:** Carlotta Nedbal, Vineet Gauhar, Shilpa Gite, Het Sevalia, Ratan Maurya, Prisha Jaiswal, Khushi Kashyap, Andrea Gregori, Francesco Antomarchi, Frédéric Panthier, Yiloren Tanidir, Abhishek Singh, Boyke Soebhali, Hsiang Ying Lee, Steffi Kar Kei Yuen, Ee Jean Lim, Nitesh Naik, Bhaskar Kumar Somani

**Affiliations:** 1 Polytechnic University Le Marche, Ancona, Italy; 2 IRCCS San Gerardo dei Tintori, Monza, Italy; 3 Endourology Section, European Association of Urology, Arnhem, The Netherlands; 4 Ng Teng Fong General Hospital, Urology, Singapore, Singapore; 5 Symbiosis Institute of Technology, Symbiosis International (Deemed University), Pune, India; 6 Tenon Hospital, Sorbonne University, GRC Urolithiasis No. 20, Paris, France; 7 PIMM, UMR 8006 CNRS-Arts et Métiers ParisTech, Paris, France; 8 Department of Urology, Marmara University School of Medicine, Istanbul, Turkey; 9 Muljibhai Patel Urological Hospital, Nadiad, Gujarat India; 10 Department of Urology, Abdul Wahab Sjahranie Hospital Medical Faculty, Muliawarman University, Samarinda, Indonesia; 11 Department of Urology, School of Medicine, College of Medicine, Kaohsiung Medical University, Kaohsiung, Taiwan; 12 Department of Surgery, SH Ho Urology Centre, The Chinese University of Hong Kong, Shatin, Hong Kong; 13 Department of Urology, Singapore General Hospital, Singapore, Singapore; 14 Department of Mechanical and Industrial Engineering, Manipal Institute of Technology, Manipal Academy of Higher Education, Manipal, 57614 Karnataka India; 15 University Hospital Southampton NHS Foundation Trust, Southampton, UK

**Keywords:** Paediatric urology, Urolithiasis, Lower pole stones, Machine learning, Predictive tools, Stone free rates.

## Abstract

**Introduction:**

The rising incidence of kidney stone disease in children presents growing clinical challenges, particularly in managing lower pole (LP) calculi, which are anatomically difficult to treat. Flexible ureteroscopy with laser lithotripsy (fURSL) has emerged as a preferred minimally invasive treatment. However, surgical outcomes remain variable, especially in the paediatric LP stone cohort. This study aimed to apply machine learning (ML) techniques to predict surgical outcomes based on preoperative characteristics and identify key predictors of incomplete stone clearance.

**Materials and methods:**

A retrospective analysis was conducted on paediatric patients (≤ 16 years) who underwent fURSL between January 2017 and December 2021 across eight tertiary centres. From a multicentre database of 280 patients, 91 with isolated LP stones were selected. Preoperative, intraoperative, and postoperative variables were analysed. Fifteen ML models—including ensemble algorithms and a multitask neural network—were developed to predict LP stone presence and postoperative outcomes. Model performance was evaluated using accuracy, precision, recall, F1-score, and SHAP (SHapley Additive exPlanations) values for interpretability.

**Results:**

LP stones were present in 32.5% of cases and were associated with older age, solitary stones, and higher stone burden. Random Forest outperformed all other models (validation accuracy: 80.95%; F1-score: 76.67%), followed by Gradient Boosting. SHAP analysis identified stone number, total stone burden, age, and operative time as top predictors. LP stones were associated with a higher rate of residual fragments (RF) and lower need for preoperative stenting or ureteral access sheath use. Infectious and bleeding complications were less frequent in the LP group.

**Conclusion:**

fURSL is safe and effective in children with LP stones, though incomplete stone clearance remains a challenge. ML models demonstrated strong predictive performance and could support preoperative risk stratification. Further external validation and prospective studies are warranted to refine predictive tools for clinical use.

**Supplementary Information:**

The online version contains supplementary material available at 10.1007/s00345-025-06095-1.

## Introduction

 Kidney stone disease is an increasingly recognised condition in the paediatric population, now affecting up to 2% of children across Europe. This rising incidence is largely attributed to lifestyle changes and a growing prevalence of malnutrition-related conditions such as obesity and metabolic syndrome [1]. Early identification, effective management, and structured follow-up are crucial to prevent recurrence. However, the diagnosis and treatment of paediatric urolithiasis present unique clinical challenges.

According to the current European Association of Urology (EAU) guidelines, different treatment modalities, similarly to the adult population, can be proposed and have been proved safe in children, encompassing flexible ureteroscopy with laser lithotripsy (fURSL), percutaneous nephrolithotomy (PCNL) and shockwave lithotripsy (SWL) [2, 3]. If historically PCNL has generally been reserved for larger stones, recent advancements such as the miniaturization of endoscopic instruments, enhanced laser technologies, and the integration of suction systems have enabled successful treatment of larger calculi using less invasive techniques [4].

fURSL has emerged as a preferred minimally invasive approach, with good safety and efficacy profile. Some debates still remain on the best approach with complex stones, and in particular for lower pole (LP) calculi. Despite the known advantages of fURSL, with lower complication rates and wider applicability compared to PCNL [5], treatment outcomes remain highly variable, influenced by both anatomical complexities and preoperative factors, areas that are the focus of ongoing research [6]. Among stone locations, LP calculi remain the most technically challenging to manage via fURSL. This difficulty arises from unfavourable anatomy, particularly the steep infundibular pelvic angle (IPA), which impairs access and hinders complete fragmentation. As a result, paediatric patients with lower pole stones often face longer, more complex procedures and lower stone-free rates (SFR) [7].

Although technological progress has improved procedural capabilities, real-world data suggest that achieving complete stone clearance remains difficult [8, 9]. To better predict surgical outcomes, nomograms have been developed to estimate SFR in paediatric patients, incorporating variables such as stone burden, location, infection status, and individual anatomy [10].

Machine learning (ML) offers significant potential in this context, enabling the analysis of complex, multidimensional data to identify predictive patterns that traditional statistical approaches may overlook [11, 12]. Complementing this, Explainable Artificial Intelligence (XAI) tools are increasingly used to improve transparency, interpretability, and clinical trust in ML-driven predictions, thereby supporting more informed and safer decision-making.

The present study retrospectively evaluated paediatric patients undergoing fURSL for isolated lower pole stones and applied ML techniques to predict surgical outcomes based on preoperative characteristics. By identifying key predictors, our aim is to facilitate individualized risk stratification and enhance surgical planning in this vulnerable population.

## Materials and methods

### Patient selection, data collection, and operative protocols

A retrospective review was performed on paediatric patients (≤ 16 years) who underwent fURSL for urolithiasis between January 2017 and December 2021 across eight high-volume tertiary centres. Data were extracted from a previously published multicentric database comprising more than 300 patients [7]. For this study, patients presenting with isolated lower pole stones were selected, resulting in a final cohort of 91 patients, and compared with the non-LP cohort.

Demographic and clinical variables collected included age, sex, comorbidities, stone burden (single vs. multiple), and maximum stone diameter. The presence of known metabolic or genetic disorders such as renal tubular acidosis (RTA), hypercalciuria, cystinuria, or hyperoxaluria/hypocitraturia was recorded. Additional variables included presenting symptoms, history of stone recurrence, and prior interventions.

Preoperative imaging modalities followed institutional protocols and included non-contrast or contrast-enhanced computed tomography (CT) and/or dedicated renal ultrasonography (USS). Patients were excluded if they had non-urolithiasis diagnoses (e.g., upper tract malignancies), anatomical renal anomalies, stones in non-lower-pole locations, were aged > 16 years, or lacked consent for data usage. Informed consent was obtained from all patients and/or legal guardians following specialist counselling. A sterile urine culture was required prior to surgery.

Intraoperative data included operative time, use of a ureteral access sheath (UAS), type of ureteroscope (reusable or single-use), laser type, and postoperative ureteral stenting. Postoperative variables included haematuria, fever, sepsis, presence and number of residual fragments (RF), and requirement for reintervention. Stone-free status was defined as no RF or RF < 2 mm on low-dose CT-KUB or USS at 3-month follow-up.

### Machine learning model development and analysis

Following data preprocessing, which included removal of irrelevant symbols and imputation of missing categorical data using mode values, statistical evaluations were conducted. These included correlation analyses, Variance Inflation Factor (ViF) assessments, and logistic regression across four predefined outcome prediction tasks.

A total of fifteen ML models were developed and individually trained to predict clinical outcomes using preoperative variables. The models encompassed multiple algorithmic categories:

- Probabilistic Methods: Naive Bayes, Logistic Regression.
- Instance-Based Learning: K-Nearest Neighbors (KNN).
- Discriminant Analysis: Linear (LDA) and Quadratic Discriminant Analysis (QDA).
- Support Vector Machines (SVM): Polynomial and RBF kernels.
- Tree-Based Algorithms: Decision Tree, Random Forest, Extra Trees, Bagging Classifier, Gradient Boosting, CatBoost, XGBoost, AdaBoost.

Additionally, a multitask Artificial Neural Network (ANN) was developed to simultaneously predict all target outcomes. The ANN architecture consisted of shared hidden layers for feature extraction and individual task-specific output layers. Rectified Linear Unit (ReLU) activations were employed in hidden layers, and sigmoid activations were used in the output layers.

Model performance was evaluated using classification reports and confusion matrices, including accuracy, precision, recall, and F1-score. To improve interpretability of complex models (e.g., ANN), explainable AI (XAI) techniques, specifically SHAP (SHapley Additive exPlanations) values were employed to highlight the most influential features in model predictions.

### Model evaluation and performance metrics

Bar charts were generated to compare model validation accuracy across various feature subsets (e.g., patient demographics, stone characteristics, metabolic parameters). Validation accuracy represented model generalizability on unseen data. Autologger was used to ensure automatic tracking of model parameters, enabling transparent and reproducible evaluation. Feature groups with higher validation accuracy were interpreted as having greater predictive value.

Additional bar plots presented training accuracies and loss metrics to assess learning behaviour across different models and feature sets. Training accuracy served to evaluate how well models fit the training data. These metrics helped identify potential overfitting when training accuracy was high, but validation performance was suboptimal. Autologger also recorded training time and loss functions to compare algorithmic efficiency and convergence.

Confusion matrices were employed to visualise classification performance by mapping predicted versus actual outcomes for each model, aiding in the identification of false positives and negatives. Each model’s classification report included key performance indicators: Precision: proportion of positive predictions that were correct; Recall: proportion of actual positives correctly identified; F1-score: harmonic mean of precision and recall; Accuracy: overall proportion of correct predictions.

## Results

### Descriptive and statistical findings

**Fig. 1:**
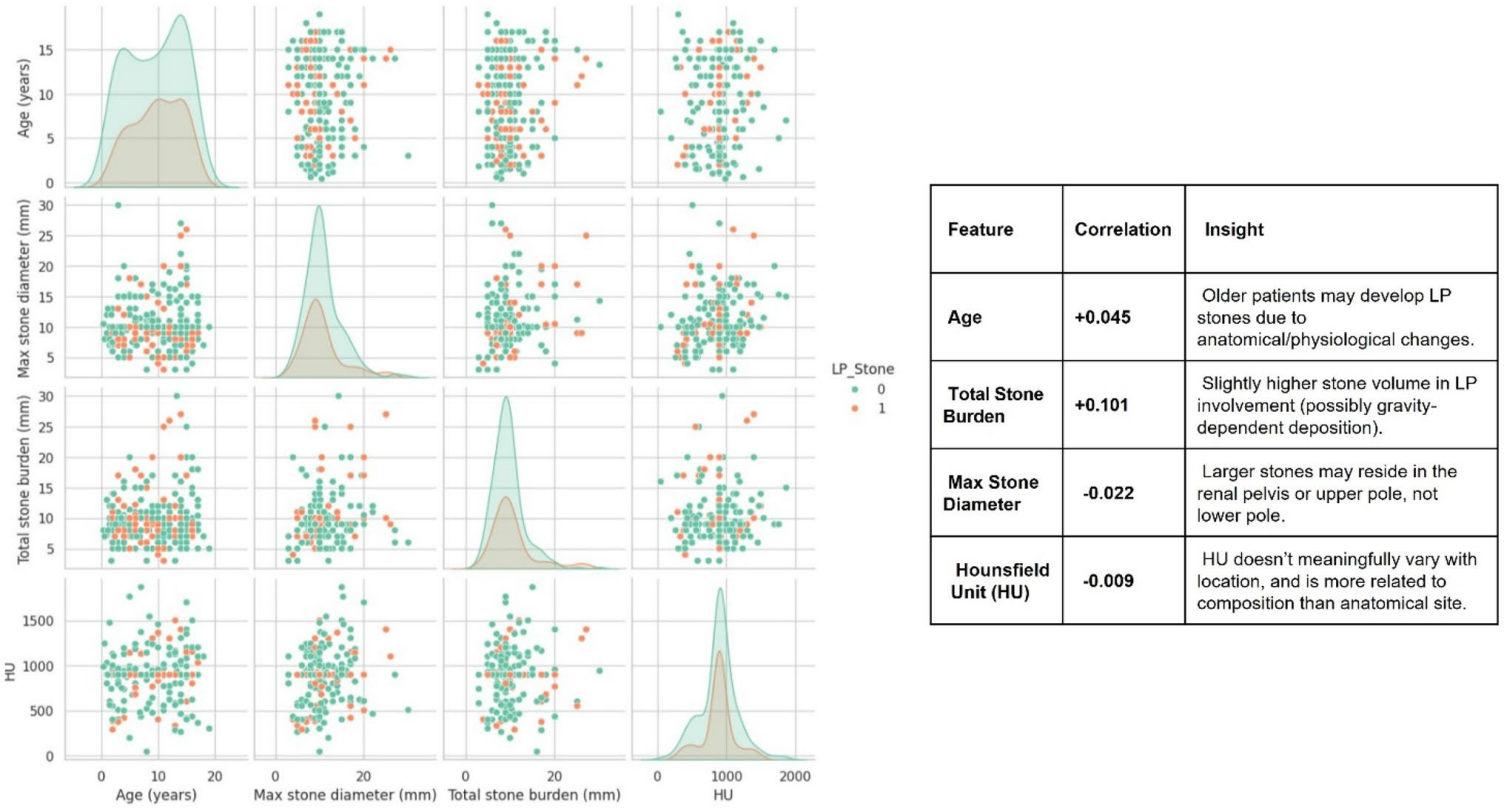
Pairwise Feature Comparison By LP Stone

A total of 280 paediatric patients were analysed, of whom 91 (32.5%) had stones located exclusively in the LP. The dataset comprised 29 variables, encompassing demographic, clinical, imaging, and procedural data. Imputation was applied for 499 missing values (numerical variables via median; categorical via mode). Comparing LP stones with the control cohort, patients affected by LP stones appeared to be slightly older, and presenting with higher total stone burden but not larger single-stone diameter (Fig. 1).

### Correlation analysis

**Fig. 2:**
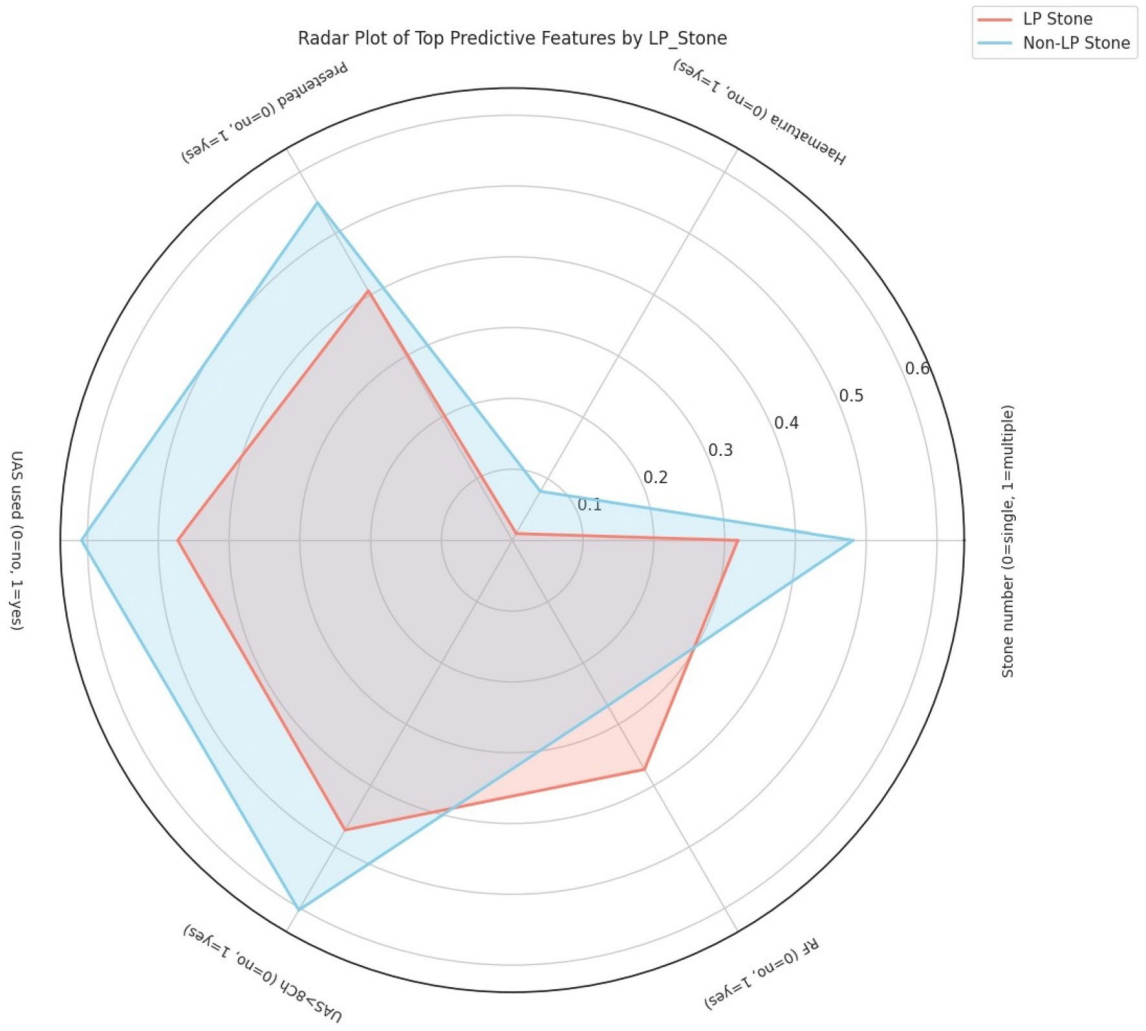
The radar plot visualizes the normalized mean values of the top predictive features that distinguish patients with LP stones (red) from those with non-LP stones (blue). This multi-feature representation enables side-by-side comparison of clinical patterns, aiding in the identification of relevant trends associated with LP stone presence

Correlation analysis revealed that no single variable exhibited strong linear association with LP stone presence (Table 1). The most positively correlated features were residual fragments (RF; r = + 0.106), total stone burden (r = + 0.101), and age (r = + 0.045). These results suggest that LP stones are more common in slightly older patients and in those with greater stone volume but are also related to a higher risk of incomplete clearance and RF, compared to the general population. In contrast, the presence of multiple stones (*r* = − 0.154), haematuria (*r* = − 0.138), pre-stenting (*r* = − 0.135), and use of a ureteral access sheath (UAS; *r* = − 0.128) were negatively associated with LP stones. This implies that LP stones tend to be solitary, less symptomatic, and can be often managed without UAS or prior stenting (Fig. 2).

There was no significant difference in stone density between the two groups, due to the fact that HU are mostly determined by stone composition and not stone location.

Postoperative complications did not show significant patterns linked with the presence of LP stones. Infectious complications like fever (*r* = -0.091) and sepsis (*r* = -0.072) showed only mild negative correlation with LP stones, and similar results were found for the presence of positive preoperative MSU (*r* = -0.054).

**Table 1:** Main results of correlation analysis, divided in negative and positive correlations (with relative r values)

Positive correlations		Negative correlations	
Feature	Correlation	Feature	Correlation
RF	+ 0.106	Stone number	−0.154
Total stone burden (mm)	+ 0.101	Haematuria	−0.138
Reintervention	+ 0.047	Presented	−0.135
Age (years)	+ 0.045	UAS used	−0.128
HolmLP	+ 0.044	Laser time (minutes)	−0.076
Reusable scope	+ 0.040	Fever	−0.091
Known genetic disorders	+ 0.038	Sepsis	−0.072
Postoperative stent	+ 0.034	Positive MSU	−0.054
Normal kidney anatomy	+ 0.012	Multiple fragments	−0.032

A hierarchical clustering analysis identified feature groups with tight interdependencies, notably between UAS use and sheath size (VIF > 70), warranting the removal of one to reduce multicollinearity. Pairwise comparisons confirmed that LP stones were more frequently associated with single stones, lower haematuria rates, and reduced use of UAS and larger sheaths.

A detailed overview of the correlation and ML analysis in available in the supplementary materials section.

### Machine learning model performance

Fifteen machine learning classifiers were trained using preoperative variables to predict the presence of LP stones. Ensemble-based models significantly outperformed individual classifiers.

Random Forest emerged as the top-performing model with a validation accuracy of 80.95%, precision of 80.63%, recall of 75.00%, and F1-score of 76.67%, reflecting excellent balance between sensitivity and specificity. Gradient Boosting demonstrated similarly robust generalization with 76.19% validation accuracy and balanced metric profiles (training accuracy: 97.06%). Extra Trees Classifier reached 73.81% validation accuracy but showed signs of overfitting due to perfect training performance.

Other strong models included CatBoost and Bagging Classifier, both achieving 71.43% validation accuracy. In contrast, Quadratic Discriminant Analysis (QDA) and Naïve Bayes underperformed (validation accuracy ~ 40–50%), while Support Vector Machine variants displayed modest accuracy (~ 66.67%) but low F1-scores, indicating poor class boundary definition.

### Feature importance and explainability

Explainable AI techniques, including SHAP (SHapley Additive exPlanations) and decision tree visualizations, provided interpretability of model predictions.

Top predictors of LP stone presence included: Stone number (single vs. multiple); Total operative time; Maximum stone diameter; Total stone burden; Age; Residual Fragments; UAS usage; and Presentation status (pre-stenting).

SHAP summary plots indicated that multiple stones most consistently increased the model’s likelihood of predicting LP stones, and this stone location was linked to shorter laser times and higher risk of RF. Conversely, features such as female sex, avoiding UAS use, and shorter operative time contributed negatively, reducing LP stone probability in specific predictions.

Decision tree visualizations confirmed that stone number was the initial node in model splitting, with subsequent branches involving RF, stone diameter, and fragmentation patterns. This hierarchy supported the clinical assumption that solitary stones and minimal instrumentation are more typical in LP stone cases.

**Fig. 3:**
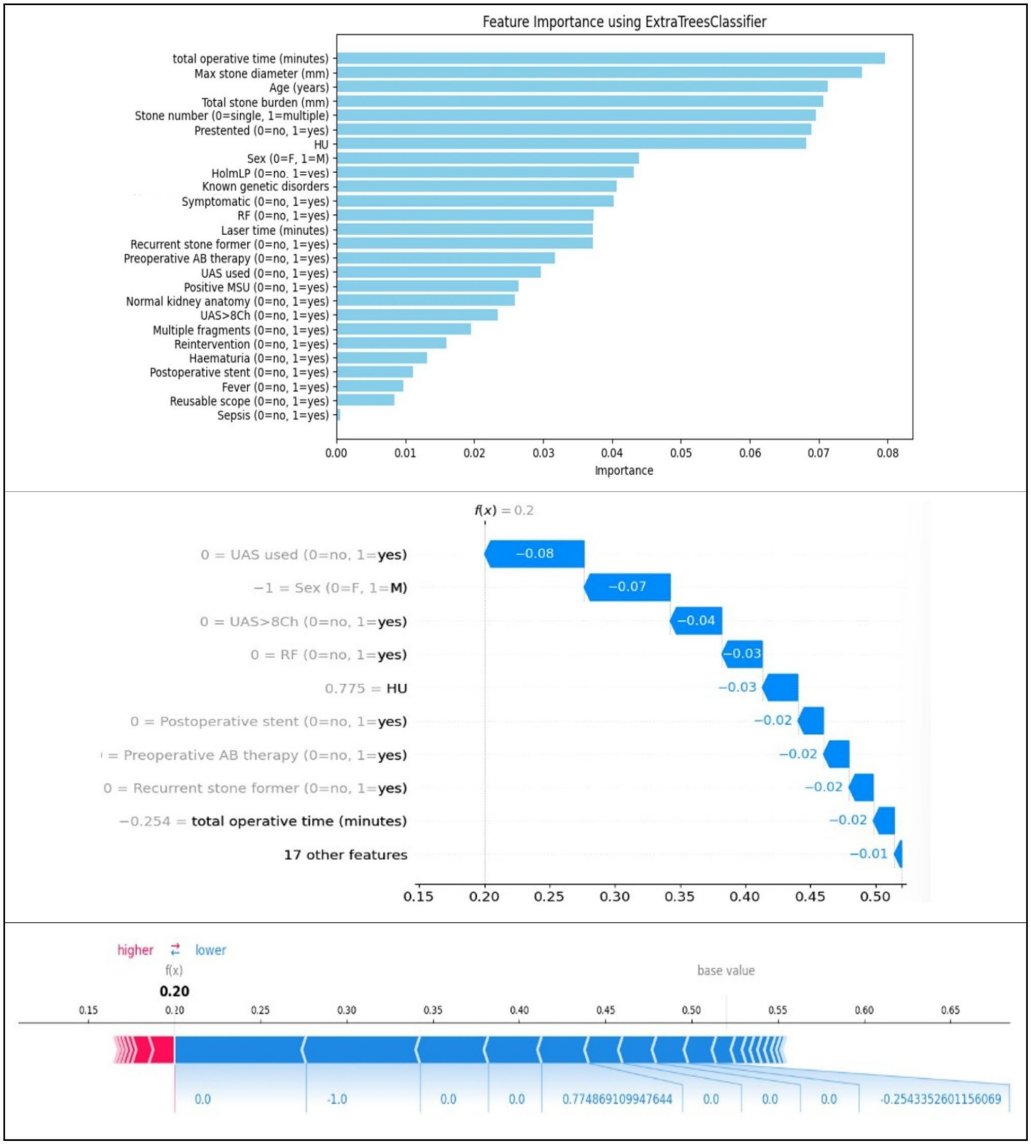
Explainable AI for LP stones. From the top: Feature Importance, SHAP Waterfall Plot (Detailed Feature Decomposition for One Prediction), SHAP Force Plot (Prediction-Level Impact Visualization)

Prediction-level explanations (via SHAP waterfall and force plots) confirmed that individual outcomes were strongly driven by procedural details and stone configuration, rather than isolated demographic or biochemical features (Fig. 3).

### Summary of predictive accuracy

Overall, ensemble models, especially Random Forest and Gradient Boosting demonstrated superior performance in identifying LP stones, with validation accuracies exceeding 75%. While individual predictors showed weak linear correlation with LP stone presence, their collective interaction in nonlinear models yielded clinically meaningful predictive power. These results highlight the value of ML in supporting diagnostic decision-making for paediatric urolithiasis.

## Discussion

The ML model trained well on the retrospective database, providing good accuracy in prediction of LP related features and outcomes. To our knowledge, this is currently the largest ML analysis on paediatric patients undergoing fURSL, and the only one to investigate the role of LP stones in probability of stone clearance and risk of complications.

As suspected, we found an increased risk of incomplete clearance in this population, alongside the need for multiple intervention in light of both the risk of residual fragments and recurrence of stones. Interestingly, infectious complications appear to be slightly less frequent in children with LP stones compared to other stone locations, and this is reflected by the lower rates of positive urine cultures and patients needing extended preoperative antibiotics. Postoperative haematuria was also less frequently encountered in this population group, despite the well-known challenges that a stiff infundibular angle can pose while treating LP stones [13]. In our opinion, these findings might somehow be related, given the increased risk of contact-bleeding and mucosal abrasion during laser treatment when dealing with inflamed tissues, characteristics of patients with recurrent urinary tract infections or infected stones.

Based on our analysis, preoperative presentations and stone characteristics can differ in children affected by LP and non-LP stones. Older children and adolescents tend to have larger stones located in the LP, but they often present with single stones that are not obstructive and therefore require preoperative stenting less frequently. These findings may relate with renal anatomy, with gravity and infundibular angle playing a role in the larger growth of urolithiasis in LP stones before they get symptomatic and prompt interventions. Similarly, we found a moderate correlation between LP stones and known genetic disorders, with increased risk of urine stasis and stone formation. This link appears to be stronger that the one with anatomical variants of the kidney.

Previous studies, not performed with the innovative aid of ML analysis, found an increased risk for incomplete clearance in children with LP stones. These results are in line with our analysis, with same risk of operative and postoperative complications reported in the two subgroups [14]. As also described by Sen and colleagues, LP stones can be difficult to completely remove, but RF in these cases can frequently be asymptomatic and more rarely require reintervention [15]. Nevertheless, thanks to technological advancements and miniaturisation of fURSL instruments, ureteroscopy can now considered a first line treatment in children with LP stones, and a two-step procedure can still be offered as complication rates and impact on children quality of life appears to be improved compared to PCNL [16]. Previous systematic review looking at the role of fURSL for paediatric renal stones recommends that these procedures are done by experienced surgeons [17].

ML has already been proposed to evaluate fURSL results, in several studies conducted in adult populations [18]. As LP stones represent a challenge also in adults, the use of suction devices has been investigated and proven safe and effective [19]. Results appears to be in line with the ones presented in this study, showing increased risk of residual fragments and complications but overall good outcomes. However, it is pivotal to remember that children have some additional risks when presenting with urolithiasis, and have higher risk of recurrence and progression. Hence, a thorough surgical planning and accurate complete stone clearance is even of greater importance in this delicate population.

Our study represents one of the first ML-based analysis on a paediatric population undergoing fURSL for urolithiasis, and the first to describe findings on a large cohort with LP stones. Despite the retrospective nature, that comes with possible biases of missing or incomplete data, the analysis and ML training resulted in good performance and identification of predictive factors and outcomes. Among limitations, we recognise the retrospective nature of the study and the variability of methods in assessing RF (USS or CT KUB), that relied on the specific protocols of each centre. This multicentric database collected a large number of paediatric cases and reflects the variety of real-word practice. We believe that this represents a strength of the study, and with further external validation and prospective analysis a predictive model might be developed for this fragile subgroup with increased risk of incomplete stone clearance. Perhaps future studies should also look at the cost of fURSL procedures and adaption of ML techniques in outcome prediction for these procedures.

## Conclusion

Children with LP stones can be safely and effectively treated with fURSL, but a higher risk of incomplete stone clearance needs to be acknowledged. Obstructive stones are less frequent in patients with LP stones, and there is a reduced need for preoperative stent insertion. Complications rates appear to be similar to the general paediatric population. ML models demonstrated strong predictive performance and could support preoperative risk stratification, but further external validation and implementation with prospective data are warranted to refine predictive tools for clinical use.

## Supplementary Information

Below is the link to the electronic supplementary material.

Supplementary Material 1

## Data Availability

No datasets were generated or analysed during the current study.
