# Supplementary material for: Efficacy and safety of ureteroscopy in children with lower pole renal stones : a machine learning predictive model from the EAU section of endourology
Source: World J Urol. 2025 Nov 20;43(1):706. doi: 10.1007/s00345-025-06095-1 (PMC12634796; doi:10.1007/s00345-025-06095-1)
Supplement: Supplementary file 1 — Supplementary Material 1 [file 345_2025_6095_MOESM1_ESM.docx]

SUPPLEMENTARY MATERIALS

1. STATISTICAL ANALYSIS
   1. Basic data information
   2. Correlation analysis
   3. Further analysis
   4. Variance Inflation Factor
2. MACHINE LEARNING MODEL ANALYSIS
   1. Models development
   2. Top Performing Models
   3. Underperforming Models
   4. Best Generalization Ability
   5. Conclusion
3. EXPLAINABLE AI RESULTS
   1. Decision Tree
   2. Feature importance
   3. SHAP Waterfall Plot
   4. SHAP Force Plot (Prediction-Level Impact Visualization)
   5. SHAP Decision Plot (Cumulative Prediction Path)
   6. SHAP Summary Plot
4. STATISTICAL ANALYSIS

# Basic Data Information

# This DataFrame has 280 rows, indexed from 0 to 279. There are 29 columns, which include:

- **Demographics** (e.g., Age, Sex)
- **Clinical indicators** (e.g., Symptomatic, Positive MSU)
- **Imaging/stone data** (e.g., HU, Max stone diameter)
- **Surgical/procedural details** (e.g., UAS used, Laser time)
- **Postoperative outcomes** (e.g., Fever, Hematuria, Sepsis)

These 26 features (Excluding the constant and two other( “Others /specify)” and “Stone location (0=UP, 1=MP, 2=LP, 3=renal pelvis, 4=ureter, 5=multiple)” which was later removed) in the image were included in the initial analysis.

## It had 499 null values which were imputed (Numerical imputed with median and categorical imputed with mode).


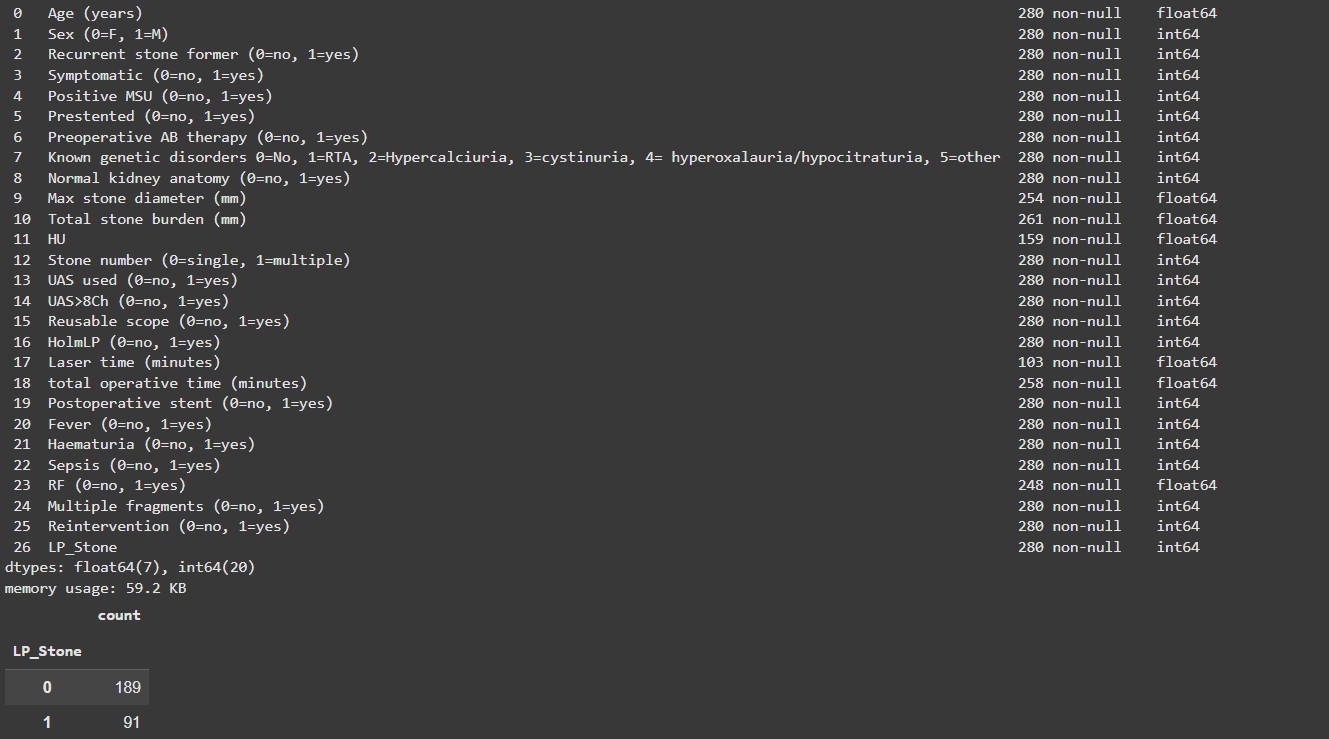


**Target variable**: LP_Stone (0 = no LP stone, 1 = LP stone)

This shows a slight class imbalance: **189 samples** have no LP stone (0); **91 samples** have LP stone (1)


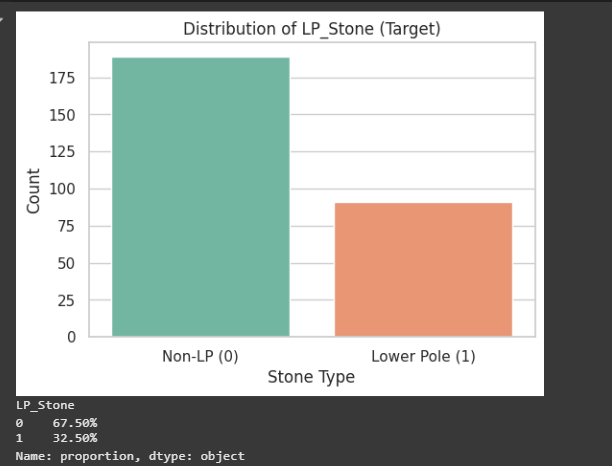


# Correlation Analysis

Focusing on how different factors are correlated with LP stone presence


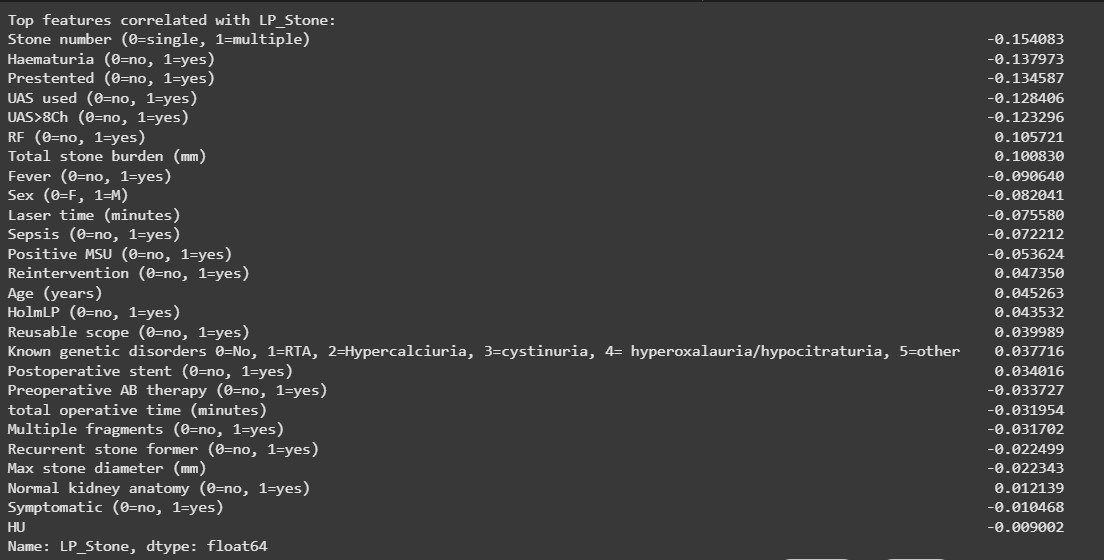


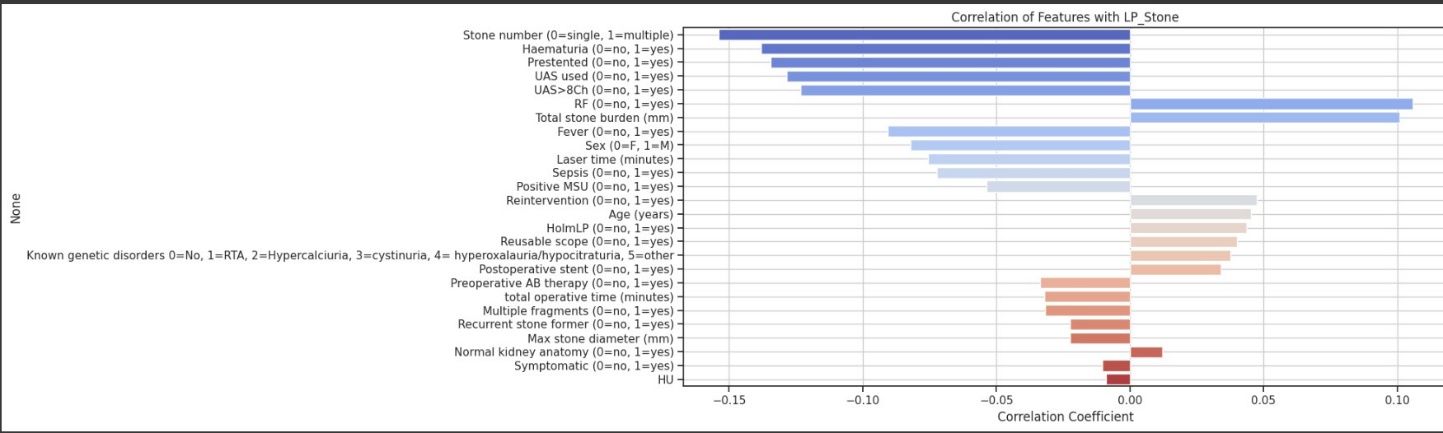


Also used a bar plot to **as it also h**elps quickly **differentiate between positively, negatively, and weakly correlated features along with the classic correlated heat map**.


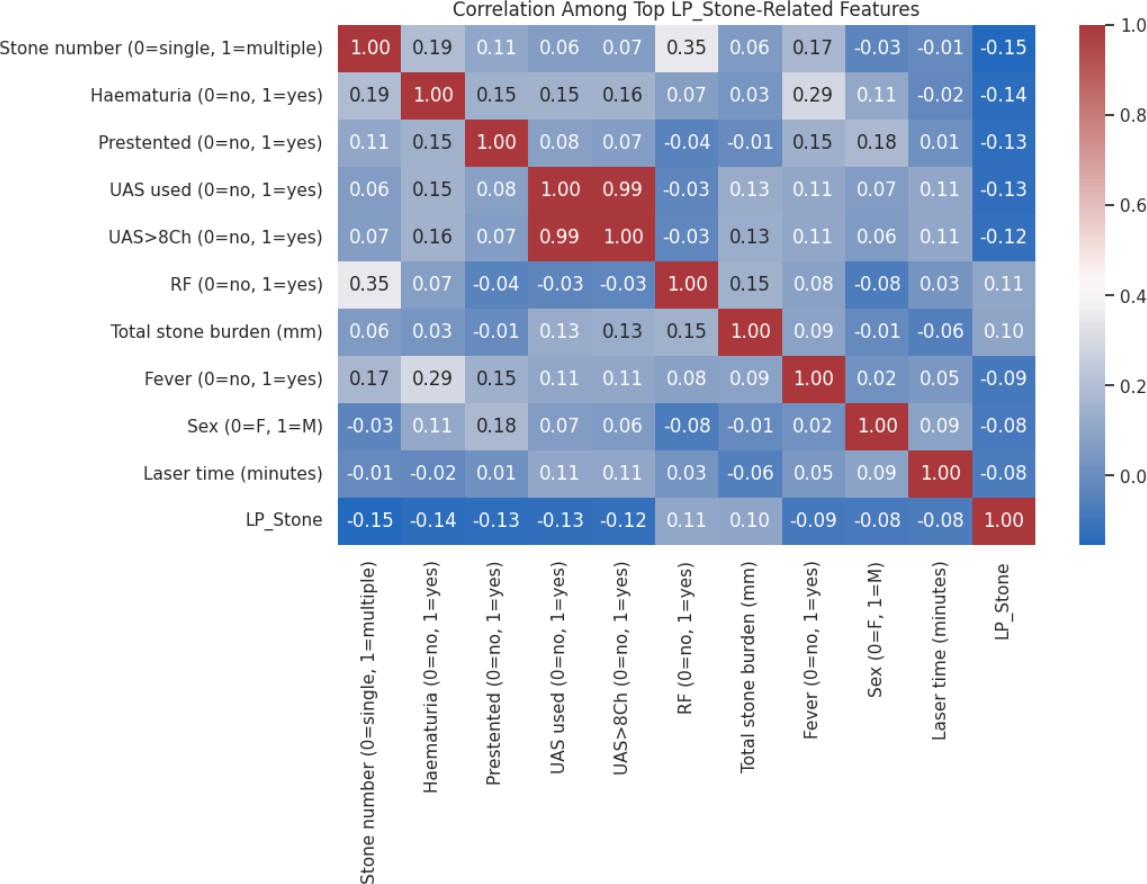


# Positive Correlations

## Higher values of these features are associated with a higher likelihood of LP stones.

| Feature | Correlation |
| --- | --- |
| RF (0=no, 1=yes) | +0.106 |
| Total stone burden (mm) | +0.101 |
| Reintervention (0=no, 1=yes) | +0.047 |
| Age (years) | +0.045 |
| HolmLP (0=no, 1=yes) | +0.044 |
| Reusable scope (0=no, 1=yes) | +0.040 |
| Known genetic disorders (0–5 categories) | +0.038 |
| Postoperative stent (0=no, 1=yes) | +0.034 |
| Normal kidney anatomy (0=no, 1=yes) | +0.012 |

RF (0.106)

→ Residual fragments may co-occur more in lower pole stones.

## Total Stone Burden (0.101)

→ Slightly higher stone volume tends to indicate LP involvement (possibly gravity-dependent deposition).


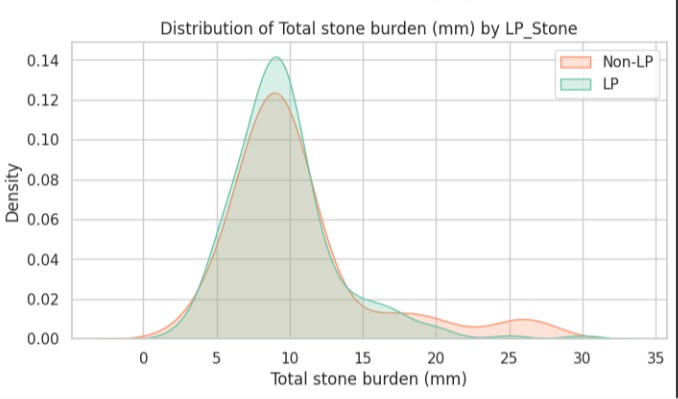


## Reintervention (0.047)

→ LP stones may have higher recurrence or be harder to completely remove.

## Age (0.045)

→ Older patients may develop LP stones due to anatomical/physiological changes.


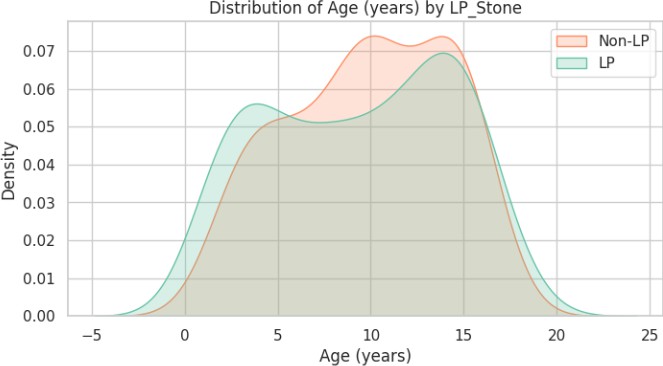


## HolmLP (0.044)

→ Use of holmium laser in LP location directly correlates with LP stone presence.

## Reusable Scope (0.040)

→ LP procedures often use reusable scopes; slight positive link.

## Known Genetic Disorders (0.038)

→ Certain stone-forming disorders may predispose to LP stones.

## Postoperative Stent (0.034)

→ Slight positive correlation, possibly due to anatomy or procedural choices.

Normal Kidney Anatomy (0.012)

→ Having normal anatomy may slightly favor LP stone development due to anatomical drainage patterns.


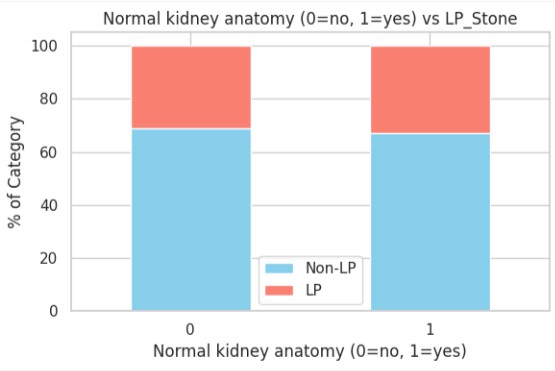


## Conclusion

Weak positive correlations suggest a slight increase in the likelihood of LP stones. **Residual freagments** (+0.106) and **total stone burden** (+0.101) were the most notable, possibly indicating that LP stones, due to drainage difficulties, might contribute to renal compromise or a significant cumulative stone burden. However, none of these are strong standalone predictors.

# Negative Correlations

## Higher values of these features are associated with a lower likelihood of LP stones.

| **Feature** | **Correlation** |
| --- | --- |
| Stone number (0=single, 1=multiple) | −0.154 |
| Haematuria (0=no, 1=yes) | −0.138 |
| Presented (0=no, 1=yes) | −0.135 |
| UAS used (0=no, 1=yes) | −0.128 |
| UAS>8Ch (0=no, 1=yes) | −0.123 |
| Fever (0=no, 1=yes) | −0.091 |
| Sex (0=F, 1=M) | −0.082 |
| Laser time (minutes) | −0.076 |
| Sepsis (0=no, 1=yes) | −0.072 |
| Positive MSU (0=no, 1=yes) | −0.054 |
| Preoperative AB therapy (0=no, 1=yes) | −0.034 |
| Total operative time (minutes) | −0.032 |
| Multiple fragments (0=no, 1=yes) | −0.032 |
| Recurrent stone former (0=no, 1=yes) | −0.022 |
| Max stone diameter (mm) | −0.022 |

Stone Number (-0.154)

→ Patients with multiple stones (1) are less likely to have a stone specifically in the lower pole.


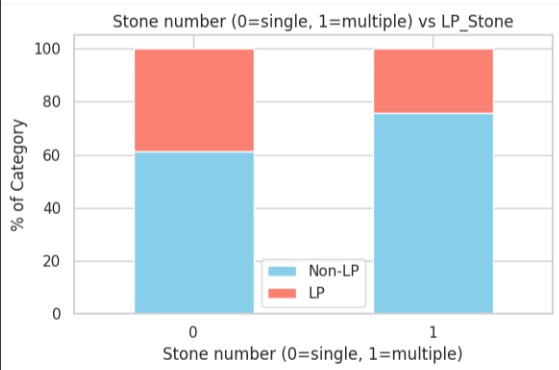


## Haematuria (-0.138)

→ Presence of blood in urine less associated with LP stones.

Presented (-0.135)

→ Patients who had stents prior to procedure are less likely to have LP stones.

## UAS Used (-0.128)

→ Use of ureteral access sheath implies more complex stone locations than LP, reducing likelihood of LP stones.

## UAS >8Ch (-0.123)

→ Larger sheath sizes typically used in non-LP stones, reducing correlation with LP stones.

## Fever (-0.091)

→ Post-op fever is slightly less associated with LP stones (maybe due to smaller size or easier retrieval).

## Sex (-0.082)

→ Males (1) are less likely to have LP stones compared to females.


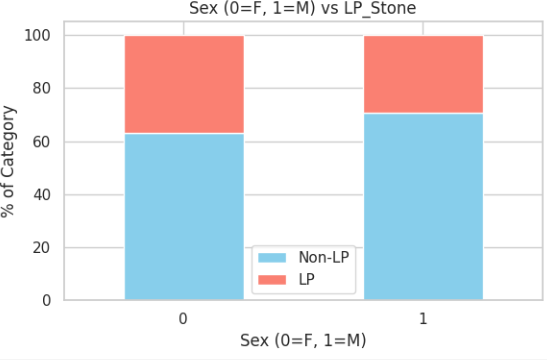


## Laser Time (-0.076)

→ Longer laser time typically correlates with complex or non-LP stones.

## Sepsis (-0.072)

→ Post-op infections (sepsis) are slightly less associated with LP stones.

## Positive MSU (-0.054)

→ Presence of bacteria in midstream urine is less correlated with LP stones.

## Preoperative AB Therapy (-0.034)

→ Pre-op antibiotics suggest infection or complex stones—not common in LP stones.


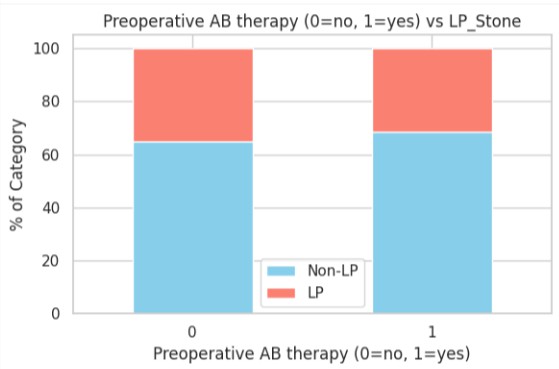


## Total Operative Time (-0.032)

→ Longer surgeries are often for **non-LP stones**.

## Multiple Fragments (-0.032)

→ Fragmentation often happens with non-LP or multiple stones, hence reduced LP correlation.

## Recurrent Stone Former (-0.022)

→ Recurrence patterns don’t strongly favor LP localization.


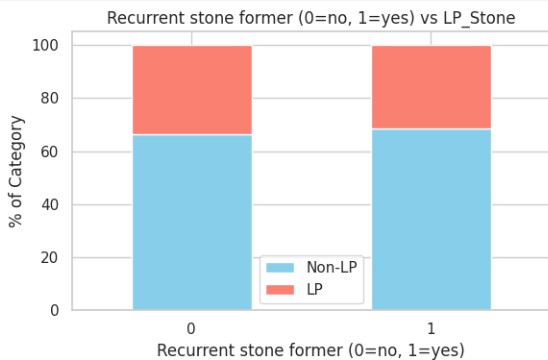


## Max Stone Diameter (-0.022)

→ Larger stones may reside in the renal pelvis or upper pole, not lower pole.


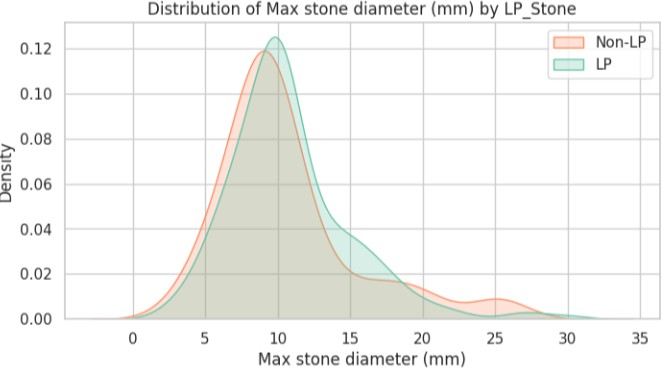


## Conclusion

Weak negative correlations suggest a reduced likelihood of LP stones. The strongest inverse relationship was with Stone number (–0.154) and haematuria (–0.138) also showed negative correlations, suggesting LP stones are often single and less frequently cause blood in urine. Pre-stented patients (–0.135) and the use of ureteral access sheath (UAS) (~–0.12) were also less likely to have LP stones.

# Weak / Minimal Correlations

## These features show very low linear correlation (near zero), indicating minimal or no direct predictive power individually.

| **Feature** | **Correlation** |
| --- | --- |
| Symptomatic (0=no, 1=yes) | −0.010 |
| HU (Hounsfield Unit) | −0.009 |
| Normal kidney anatomy (0=no, 1=yes) | +0.012 |
| Postoperative stent (0=no, 1=yes) | +0.034 |
| Known genetic disorders (0–5) | +0.038 |
| Reusable scope (0=no, 1=yes) | +0.040 |

Symptomatic (0=no, 1=yes):

- Correlation: -0.010
- Explanation: Whether the patient presents with symptoms doesn’t significantly differ between LP and non-LP stones. LP stones might be asymptomatic or mimic other locations.


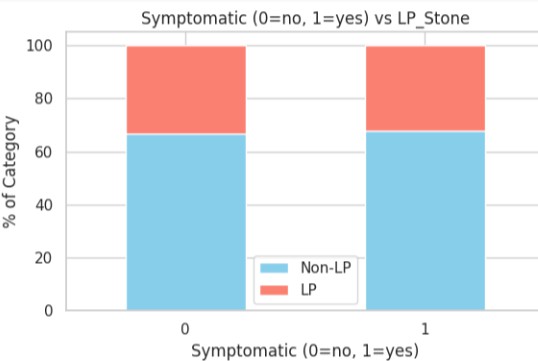


## HU (Hounsfield Unit):

- Correlation: -0.009
- Explanation: Stone radiodensity (HU) doesn’t meaningfully vary with location (LP vs. others). HU is more related to composition than anatomical site.


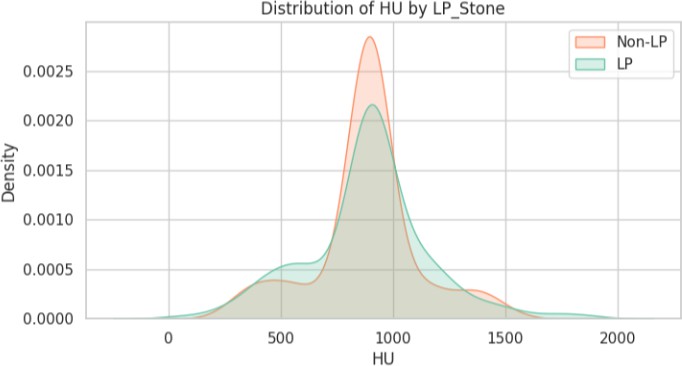


## Normal kidney anatomy (0=no, 1=yes):

- Correlation: +0.012
- Explanation: Having normal renal anatomy has a very slight positive correlation with LP stones, possibly due to typical gravitational pooling, but the effect is negligible.

## Postoperative stent (0=no, 1=yes):

- Correlation: +0.034
- Explanation: Stents are placed post-op for many stone cases regardless of location, so there's only a weak tendency toward their use in LP stones.

## Known genetic disorders (0–5):

- Correlation: +0.038
- Explanation: Genetic conditions like cystinuria or hyperoxaluria can predispose to stone formation, but distribution across anatomical locations (LP vs others) is not significantly skewed.


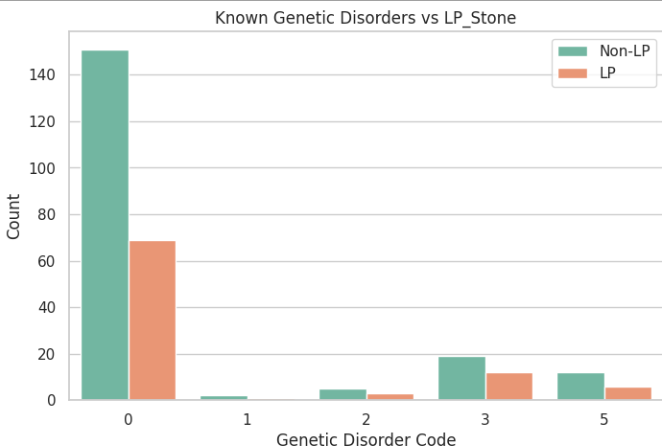


## Reusable scope (0=no, 1=yes):

- Correlation: +0.040
- Explanation: Slightly more reusable scopes might be used for LP stones, possibly due to repeated usage in confined lower calyx anatomy, but this is a procedural nuance with minimal direct effect on stone location.

## Conclusion

Most remaining variables had negligible correlations (between –0.05 and +0.05), indicating no meaningful relationship with LP stone presence. This includes positive midstream urine (MSU) culture, preoperative antibiotic therapy, total operative time, multiple fragments, maximum stone diameter, recurrent stone former status, and HU value (stone density). Even normal kidney anatomy and symptom status showed almost no correlation. These findings collectively underscore that no single variable strongly determines LP stone presence; instead, it is a subtle and multifactorial relationship.

# Pairwise Feature Comparison By LP Stone

**
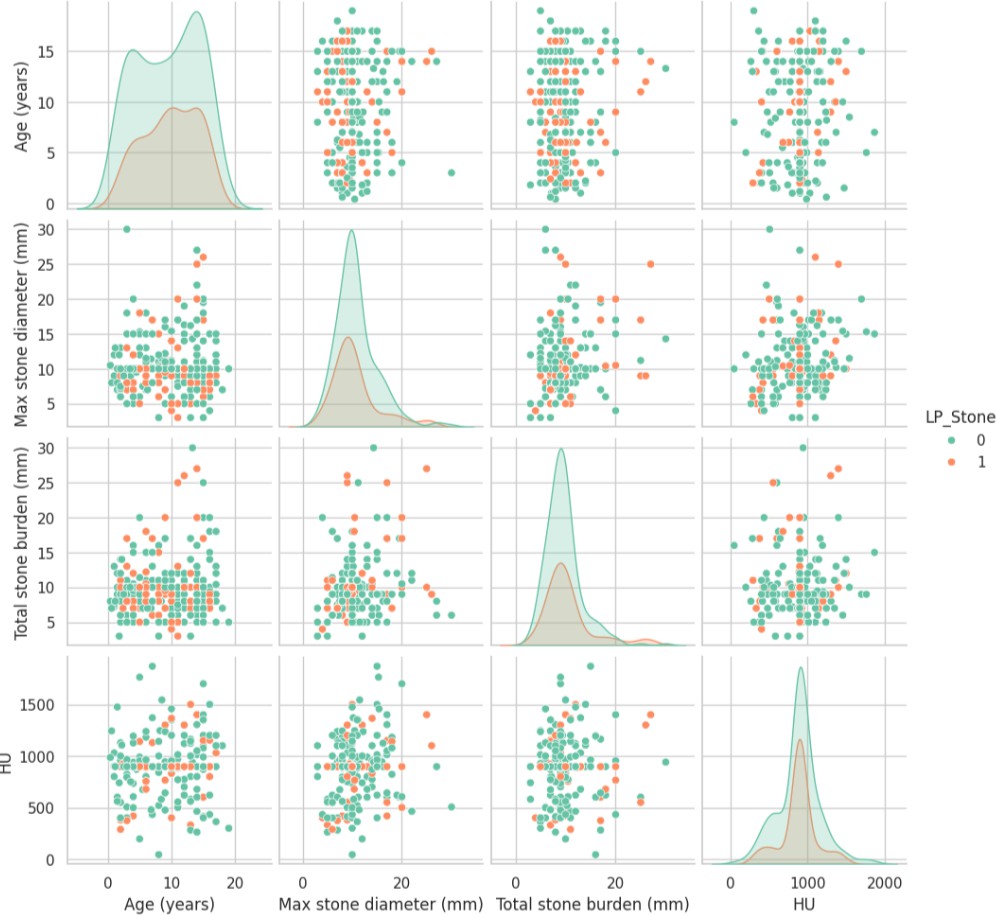
**

| Feature Pair | Insight |
| --- | --- |
| Age | LP patients skew slightly older. |
| Max stone diameter vs Burden | Correlated, may not both be necessary. |
| HU | Not class-separating; may have low predictive value. |
| Total burden | LP groups tend to have slightly lower burden. |
| Overall | No pair clearly separates classes, but patterns (e.g. lower burden, older age) help identify LP tendencies. |

## From All The features the top features used for further analysis were :

1. Stone Location

Although weakly correlated with LP_Stone, stone location directly captures anatomical sites and remains the most relevant feature. Its clinical linkage to LP diagnosis makes it indispensable, even with modest correlation.

## Stone Number

While its correlation with LP_Stone is very weak, the presence of multiple stones often implies widespread distribution, reducing the likelihood of isolated lower pole stones. It enhances model accuracy through interaction effects with other features like stone burden.

## Haematuria

Despite a weak negative correlation, haematuria remains a clinically important symptom. Its presence may signal more centrally located or obstructive stones, making it a useful discriminative feature when combined with others.

## Presented

Included for its procedural relevance, pre-stenting is often unnecessary for LP stones, hence the negative trend. Its selection is supported by its contribution to model performance, especially in combination with access-related variables.

## UAS Used

Although weakly correlated with LP_Stone, it reflects procedural complexity and is a proxy for stone location. It ranks high in feature importance due to its predictive synergy with related surgical variables.

## UAS >8Ch

Selected for its procedural context, larger sheaths are rarely used in LP stones due to access constraints. Despite high collinearity with UAS used, it contributes valuable information in models unless one is excluded to reduce redundancy. Although UAS used and UAS >8Ch show weak individual correlations with LP_Stone, they were included as top predictive features because they reflect clinically relevant procedures, interact well with other variables, and contribute meaningfully to the model’s classification performance, as seen in feature importance rankings. Their high multicollinearity with each other suggests using only one of them in modeling.

# Further Analysis

## Radar Plot of Top Predictive Features by LP_Stone

**
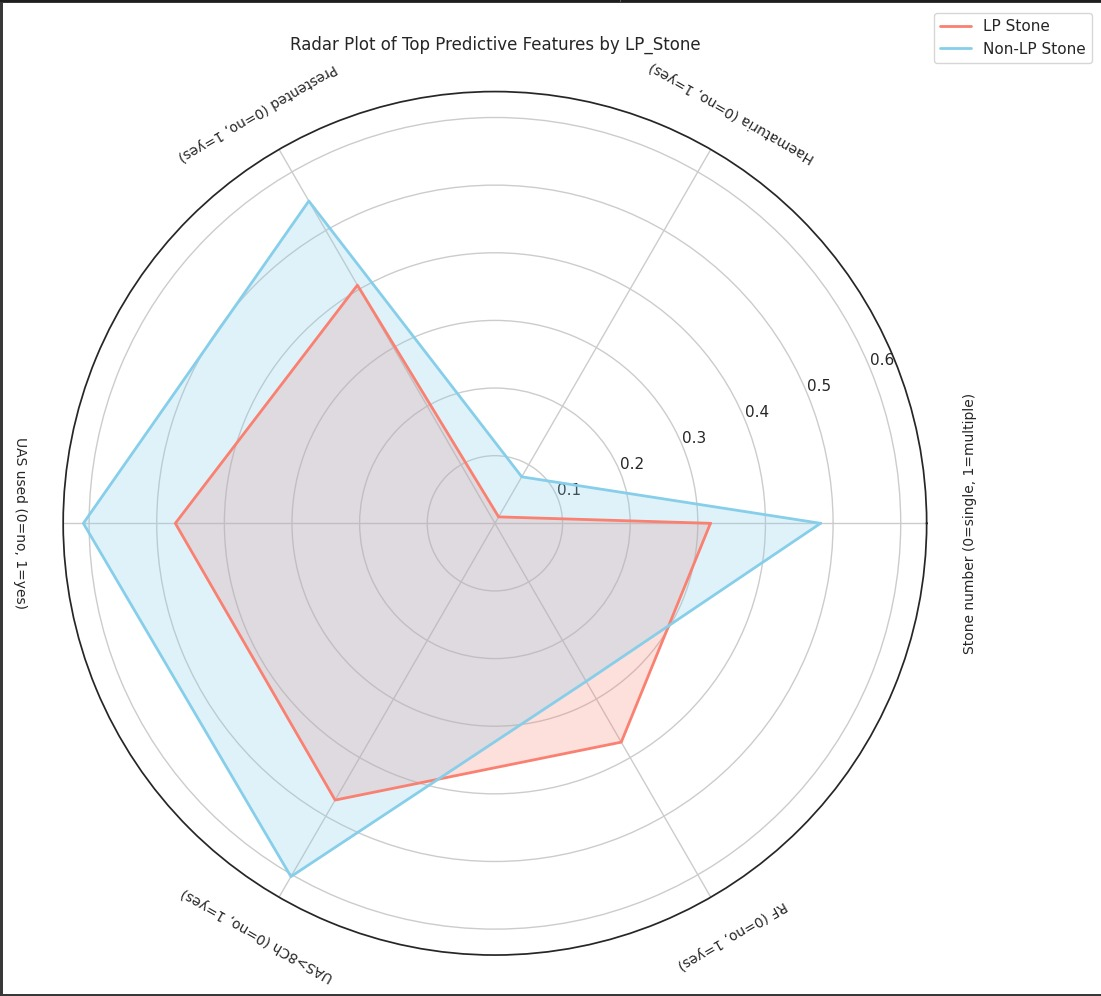
**

The radar plot visualizes the normalized mean values of the top predictive features that distinguish patients with lower pole (LP) stones (red) from those with non-LP stones (blue). This multi-feature representation enables side-by-side comparison of clinical patterns, aiding in the identification of relevant trends associated with LP stone presence.

## Feature-wise Interpretation:

- Presented (0 = no, 1 = yes): LP stone patients have a lower mean value, suggesting they are less likely to present with prior stenting, compared to non-LP cases.
- Haematuria (0 = no, 1 = yes): LP stone patients exhibit a notably lower occurrence of haematuria, potentially indicating fewer symptoms or less mucosal irritation due to stone
  - location.
- Stone Number (0 = single, 1 = multiple): LP stone cases are more likely to have a single stone, while non-LP cases are more often associated with multiple stones.
- UAS Used (0 = no, 1 = yes): The use of a ureteral access sheath (UAS) is less frequent in LP stone cases, implying that procedural access to the lower pole may be attempted without UAS more often.
- UAS > 8Ch (0 = no, 1 = yes): Similarly, the use of larger-sized UAS (>8Ch) is lower in the LP group, aligning with the above and indicating a less invasive or modified approach for LP stone management.
- RIRF (0 = no, 1 = yes): Interestingly, LP stone cases show a slightly higher rate of undergoing retrograde intrarenal surgery (RIRS), suggesting a higher need for definitive intervention in such anatomically challenging locations.

## Dendrogram heatmap


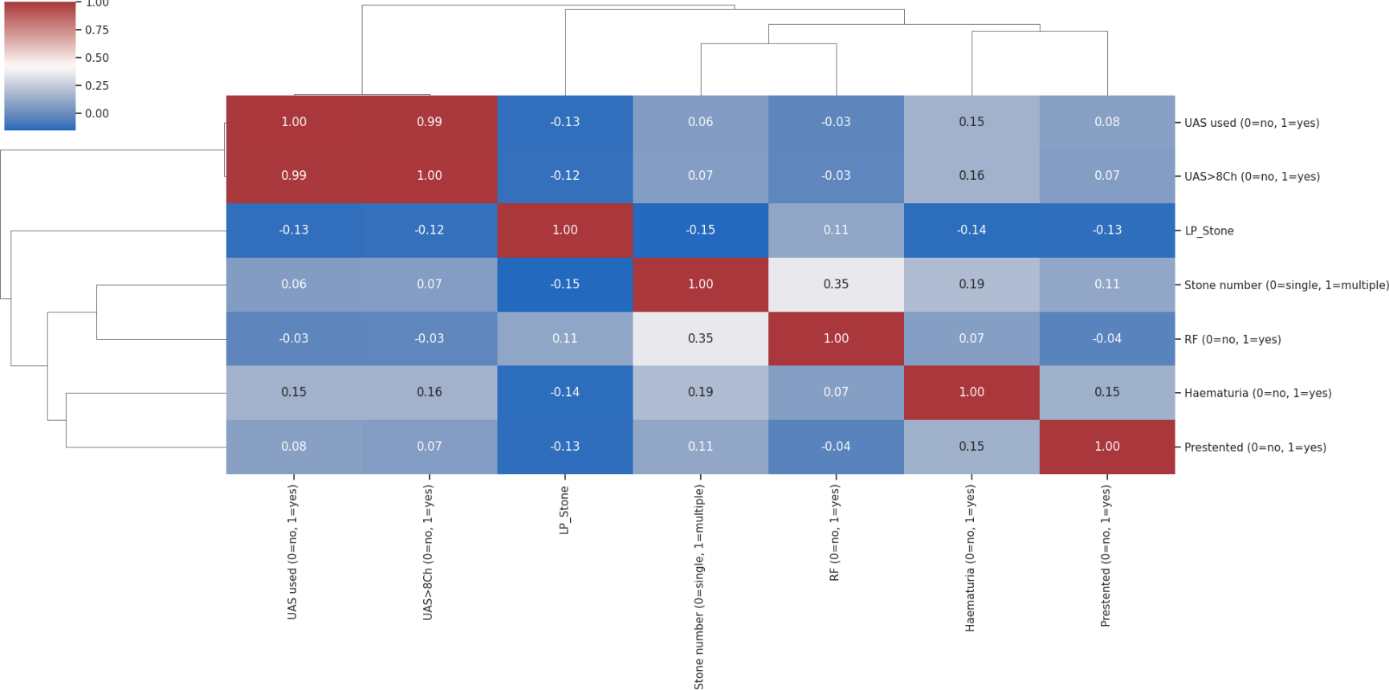


A dendrogram heatmap was utilized to visualize the hierarchical clustering of correlation values among the most influential features and the presence of lower pole (LP) kidney stones. This powerful visualization tool was chosen to thoroughly investigate the intricate relationships that exist between the variables themselves, particularly among the top predictors identified in our analysis. Furthermore, it helped us detect potential multicollinearity or feature redundancy, which is crucial for building robust statistical models. By grouping similarly behaving features, the heatmap also aids in optimizing model input.

**Key Insights from the Heatmap** **Strongest Relationships with LP Stone**

Negative Correlations (Avoidance Patterns).

LP Stone is inversely associated with:

- UAS used (r=−0.13) → Lower pole stones are less likely to involve UAS.
- UAS > BCN (r=−0.12) → If UAS is used, it’s less likely to be large ($>$BCN).
- Stone number (r=−0.15) → Lower pole stones are slightly less likely to be multiple.
- Haematuria (r=−0.14) → Lower pole stones may have less bleeding.

Interpretation: Clinicians may avoid UAS for lower pole stones due to anatomical challenges (e.g., acute infundibulopelvic angle). Single stones dominate in the lower pole (consistent with known epidemiology).

## Positive Correlation

- RF (r=0.11) → Weak link to retrograde fluoroscopy.

Possible reason: Lower pole stones may require more imaging guidance for access.

1. LP_Stone vs. Procedural Variables (UAS, RF, Presented)

| Variable | Correlation | Interpretation |
| --- | --- | --- |
| UAS used | -0.13 | Avoidance of UAS in lower pole stones. |
| UAS > BCN | -0.12 | Smaller sheaths preferred if UAS is used. |
| RF | +0.11 | Slightly higher use of fluoroscopy (likely for precise targeting). |
| Presented | -0.13 | Lower pole stones are less likely to be presented (e.g., fewer stents pre-op). |

1. LP_Stone vs. Clinical Outcomes (Haematuria, Stone Number)

| Variable | Correlation | Interpretation |
| --- | --- | --- |
| Stone number | -0.15 | Lower pole stones are more often single. |
| Haematuria | -0.14 | Less bleeding observed (possibly due to less UAS/trauma). |

1. Dendrogram Clustering (Inferred)

If visualized as a dendrogram heatmap:

- LP_Stone branches separately due to its negative correlations with UAS/stone number.
- UAS variables cluster tightly (r=0.99), far from LP_Stone.
- Stone number + RF form a subcluster with weak ties to LP_Stone.

## Parallel Coordinates Plot: Visualizing Feature Distributions


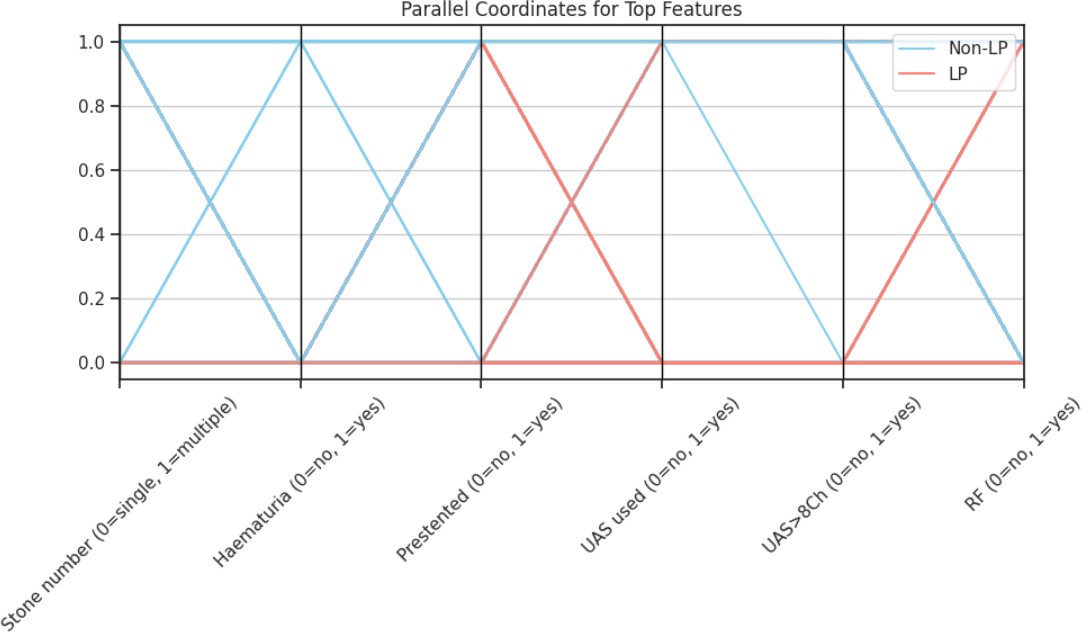


This parallel coordinate plot visualizes the relationships between various features and two stone classifications: "Non-LP" (Non-Lower Pole) and "LP" (Lower Pole). Each vertical axis represents a different feature, and lines connect the values of these features for each stone classification.

Key for the Axes:

All features are binary, represented as 0 or 1. 0 = single / no; 1 = multiple / yes

Interpretation of the Lines:

- Blue Line (Non-LP): Represents stones that are not in the lower pole.
- Red Line (LP): Represents stones that are in the lower pole.

## Analyzing Each Feature from Left to Right:

1. Stone number (0=single, 1=multiple)
   - Non-LP (Blue): Shows a value of 1, indicating that Non-LP stones are more likely to be multiple.
   - LP (Red): Shows a value of 0, indicating that LP stones are more likely to be single.

## Haematuria (0=no, 1=yes)

- - Non-LP (Blue): Shows a value of 1, indicating that Non-LP stones are more likely to present with haematuria (bleeding).
  - LP (Red): Shows a value of 0, indicating that LP stones are more likely to present with no haematuria.

## Presented (0=no, 1=yes)

- - Non-LP (Blue): Shows a value of 1, indicating that Non-LP stones are more likely to be presented.
  - LP (Red): Shows a value of 0, indicating that LP stones are more likely to be not presented.

## UAS used (0=no, 1=yes)

- - Non-LP (Blue): Shows a value of 1, indicating that UAS (Ureteral Access Sheath) is more likely to be used for Non-LP stones.
  - LP (Red): Shows a value of 0, indicating that UAS is less likely to be used for LP stones.

## UAS > 8Ch (0=no, 1=yes)

- - Non-LP (Blue): Shows a value of 1, indicating that if UAS is used for Non-LP stones, it's more likely to be a large UAS (greater than 8Ch).
  - LP (Red): Shows a value of 0, indicating that if UAS is used for LP stones, it's more likely to be a smaller UAS (not greater than 8Ch), or not used at all, consistent with the previous finding.

## RF (0=no, 1=yes)

- - Non-LP (Blue): Shows a value of 0, indicating that RF (Retrograde Fluoroscopy) is less likely to be used for Non-LP stones.
  - LP (Red): Shows a value of 1, indicating that RF is more likely to be used for LP stones.

# Variance Inflation Factor - VIF

Variance Inflation Factor (VIF) measures multicollinearity among independent variables in regression. A high VIF value (&gt;10) indicates strong correlation between variables,potentially affecting model stability.


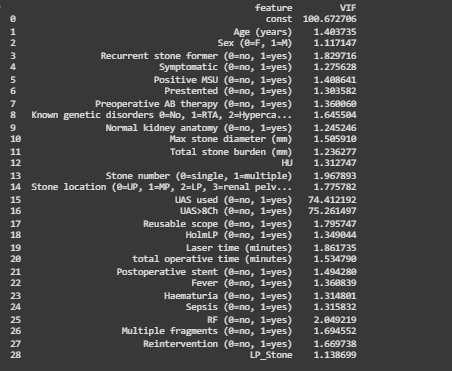


## Key Observations from the VIF Table

- High Multicollinearity:
  - UAS used (74.41) and UAS >8Ch (75.26) show extremely high VIF values.
  - Interpretation: These variables are almost perfectly correlated, causing redundant information and model instability.

## Moderate Multicollinearity (VIF 5–10):

- - Max stone diameter (5.56) and HU (5.31) fall into this range.
  - Interpretation: These might be somewhat correlated with total stone burden or other imaging metrics.

## Low/Acceptable VIF (< 5):

- - All other features have acceptable VIFs, including demographics (Age, Sex), clinical factors (Fever, Sepsis), and stone characteristics (Stone location, Stone number).
  - The target variable (LP_Stone) has no multicollinearity issue (VIF = 1.13).

1. MACHINE LEARNING MODEL ANALYSIS
2. **Models development**

**Inputs**

1. Age (years)
2. Max stone diameter (mm)
3. Total stone burden (mm)
4. HU
5. Laser time (minutes)
6. Total operative time (minutes)
7. RF (0=no, 1=yes)
8. Sex (0=F, 1=M)
9. Recurrent stone former (0=no, 1=yes)
10. Symptomatic (0=no, 1=yes)
11. Positive MSU (0=no, 1=yes)
12. Presented (0=no, 1=yes)
13. Preoperative AB therapy (0=no, 1=yes)
14. Known genetic disorders (0=No, 1=RTA, 2=Hypercalciuria, 3=cystinuria, 4= hyperoxaluria/hypocitraturia, 5=other)
15. Normal kidney anatomy (0=no, 1=yes)
16. Stone number (0=single, 1=multiple)
17. UAS used (0=no, 1=yes)
18. UAS>8Ch (0=no, 1=yes)
19. Reusable scope (0=no, 1=yes)
20. HolmLP (0=no, 1=yes)
21. Postoperative stent (0=no, 1=yes)
22. Fever (0=no, 1=yes)
23. Haematuria (0=no, 1=yes)
24. Sepsis (0=no, 1=yes)
25. Multiple fragments (0=no, 1=yes)
26. Reintervention (0=no, 1=yes)

**Output**

LP_stone (0=no, 1=yes)

# ML Algorithms:

1. naive bayes
2. knn
3. svm-linear
4. logistic regression
5. linear discriminant analysis
6. decision tree
7. bagging classifier
8. extra trees classifier
9. gradient boost
10. random forest
11. cat boost classifier
12. xgboost
13. quadratic discriminant analysis
14. svm- poly
15. svm-rbf


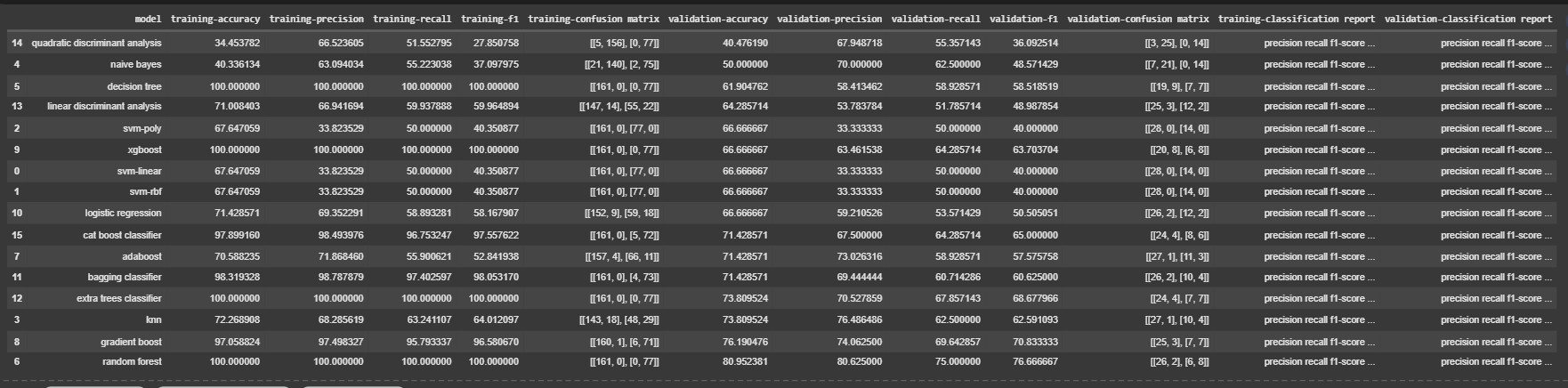


The table presents a comparative evaluation of various classification algorithms applied to a binary classification task. Each model is assessed based on training and validation performance across standard metrics: accuracy, precision, recall, F1-score, and confusion matrix. This multi-metric evaluation offers a holistic view of the models' learning capacity, bias-variance trade-offs, and generalization abilities.

# Top Performing Models

Random Forest, Extra Trees Classifier, and Gradient Boosting emerge as the most effective models. Random Forest achieves the highest validation accuracy of 80.95%, with excellent validation precision (80.63%), recall (75%), and F1-score (76.67%), alongside perfect training performance, reflecting strong generalization. Extra Trees shows perfect training accuracy (100%) and strong validation accuracy (73.81%) with balanced metrics, although the high training scores suggest a risk of overfitting.

Gradient Boost provides a good compromise, with 97.06% training accuracy and 76.19% validation accuracy, maintaining robustness across metrics and suggesting low variance and high reliability.

CatBoost and Bagging Classifier also perform reliably. CatBoost shows 71.43% validation accuracy, with good training performance and generalization. Bagging Classifier has 98.32% training accuracy and 71.43% validation accuracy, though with signs of mild overfitting.

AdaBoost achieves 71.43% validation accuracy but has a lower validation F1-score (57.57%), indicating it may benefit from further tuning.

# Underperforming Models

Quadratic Discriminant Analysis (QDA) and Naïve Bayes underperformed.

QDA achieves 40.48% validation accuracy, with low recall and F1-score, making it ineffective for this task. Naïve Bayes has 40.34% training accuracy and 50.00% validation accuracy, suggesting limited learning and poor generalization**.**

Support Vector Machines (SVM) with polynomial, linear, and RBF kernels each reach 66.67% validation accuracy, but suffer from consistently low precision, recall, and F1-scores (~40%), implying inadequate decision boundaries for this dataset.

k-Nearest Neighbors (k-NN) achieves 72.27% training accuracy, yet only 73.81% validation accuracy, and an F1-score of 62.59%, revealing signs of overfitting and sensitivity to local data fluctuations.

# Best Generalization Ability

Logistic Regression and Linear Discriminant Analysis (LDA) deliver reasonable validation accuracy (66.67% and 64.29%, respectively), while maintaining training accuracy in the 71–72% range. Their balanced performance indicates strong generalization with minimal overfitting, making them ideal for interpretable, lightweight models.

Gradient Boosting also demonstrates excellent generalization, with validation metrics that closely mirror training performance, emphasizing its adaptability and controlled complexity.

# Key Observations

- - **Ensemble models—**particularly Random Forest, Extra Trees, Gradient Boosting, and CatBoost—clearly outperform individual classifiers, benefiting from variance reduction and enhanced learning capacity.
  - **Decision Tree,** though achieving 100% training accuracy, suffers from a drop to 61.90% validation accuracy, highlighting overfitting in its raw form.
  - **SVM models with non-linear kernels (RBF and Polynomial)** consistently fail to generalize, reinforcing their unsuitability for this dataset.
  - **Linear models like Logistic Regression, LDA, and SVM-Linear** exhibit moderate but stable validation scores, supporting their use in applications favoring interpretability and generalization.

**Visual Representation for the same (training):**

**
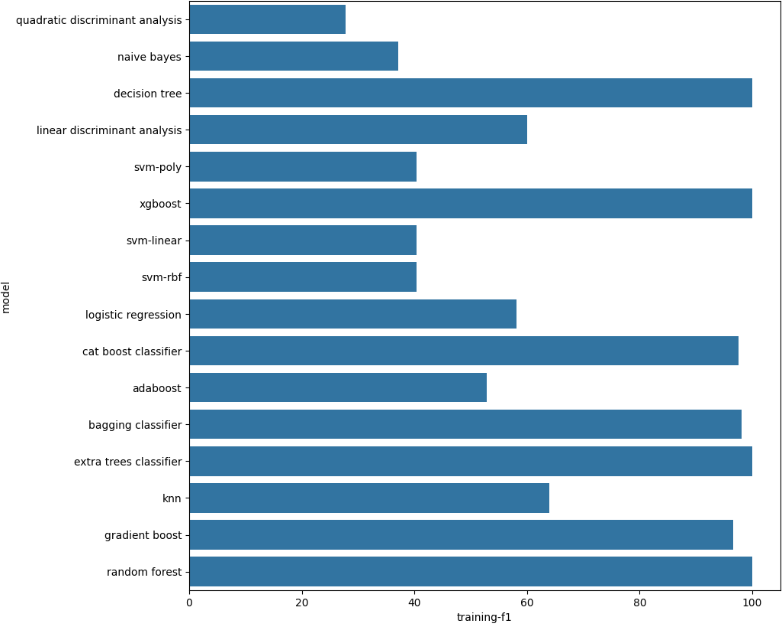
**

**Visual Representation for the same (validation):**

**
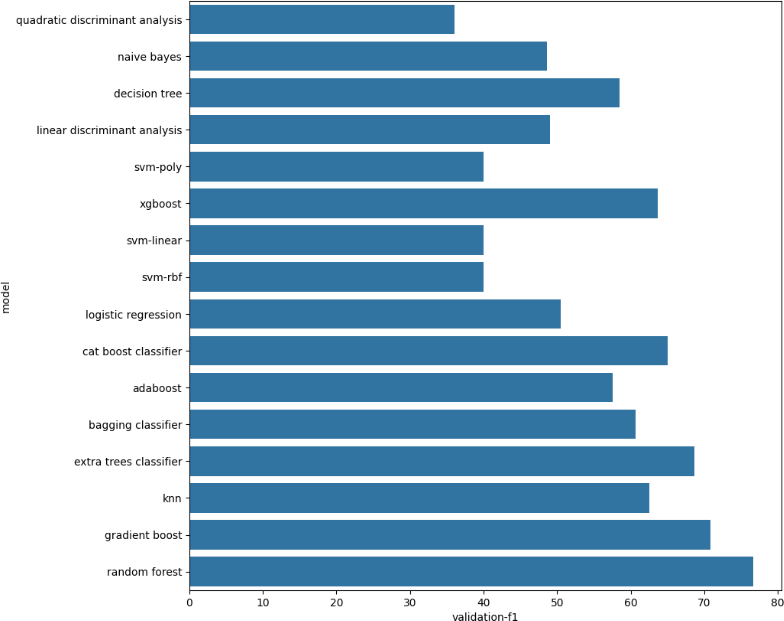
**

1. **Conclusion**

The Random Forest model is recommended as the primary diagnostic tool, demonstrating an accuracy of 80.95%. For an additional layer of validation and robustness, the Gradient Boost model, with an accuracy of 76.19%, is designated as the backup model.

For optimal diagnostic reliability, an ensemble approach is advised, utilizing both models to reach a consensus diagnosis. This strategy leverages the strengths of each model to enhance overall confidence in the results.

The models exhibit strong clinical performance. The sensitivity (recall) ranges from 69-75%, which is highly effective in identifying true positive cases. Furthermore, the high precision of the models indicates their proficiency in minimizing false positive diagnoses. The overall accuracy for medical diagnosis is excellent, ranging from 76% to 81%, signifying a reliable and effective diagnostic system.

1. EXPLAINABLE AI RESULTS
2. **Decision Tree**

**
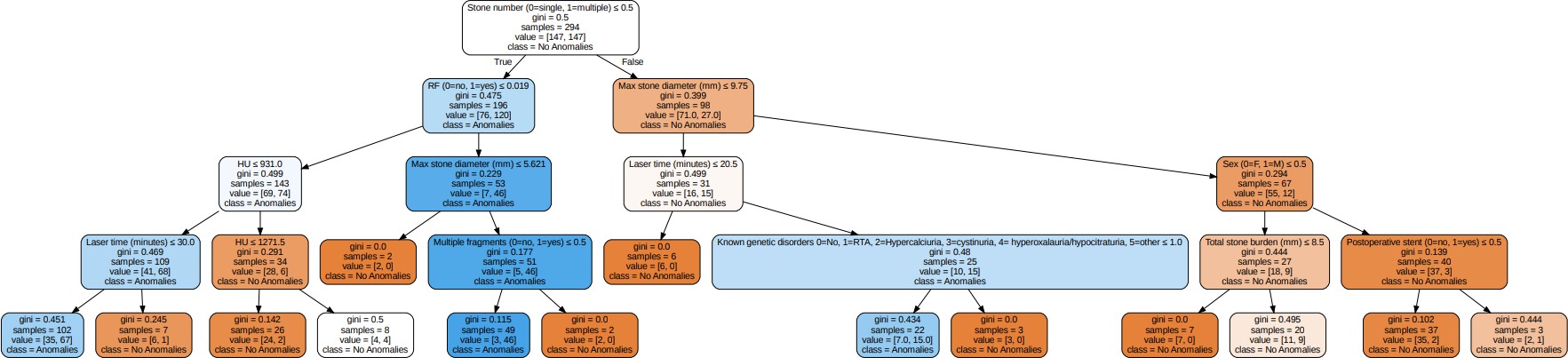
**

**Root Node (Top Node)**

- - Feature: Stone number (0=single, 1=multiple)
  - Condition: Stone number ≤ 0.5
  - Gini index: 0.5 (maximum impurity – perfectly balanced classes)
  - Samples: 294
  - Value: [147 No Anomalies, 147 Anomalies]
  - The model splits the population first based on whether the patient has a single or multiple stones.

## Decision Nodes (Intermediate Nodes)

- - Example Split:
    - Condition: RF (0=no, 1=yes)
    - Gini: 0.475
    - Samples: 126
    - Value: [65 No Anomalies, 61 Anomalies]
    - Patients with single stones are further split based on renal function status, followed by

## Max stone diameter and Multiple fragments.

- - Another Path:
    - Condition: Max stone diameter ≤ 9.75
    - Gini: 0.291

## Samples: 98

- - - This separates patients with smaller vs. larger stones, impacting classification sharply.

1. **Leaf Nodes (Bottom Nodes)**
   - Example:
     - Gini = 0.0, Samples = 2, Value = [2, 0]
     - Perfectly pure node predicting No Anomalies.
     - These nodes represent the final decision point — no further splitting.
2. **Feature Importance**

**
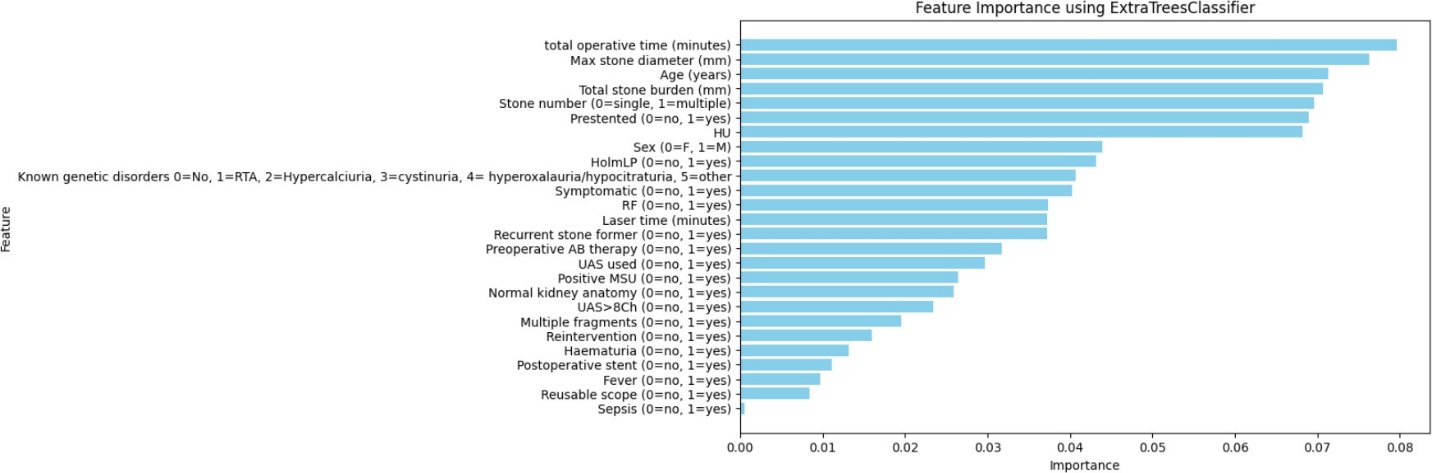
**

1. **Top Features Contributing to the Model:**

- Total operative time (minutes) is the most important feature, contributing the most to the model’s predictive ability.
- Max stone diameter (mm), Age (years), and Total stone burden (mm) are also highly significant, indicating the model heavily considers surgical complexity and patient profile.
- Stone number (0=single, 1=multiple) and Presented (0=no, 1=yes) are also strong predictors, suggesting that stone multiplicity and clinical presentation play major roles.
- HU (Hounsfield Units) and Sex also show moderate contribution.

## Moderately Important Features:

- HolmLP, Symptomatic status, RF (renal function issues), Laser time, Recurrent stone former, and Preoperative AB therapy add predictive value, but not as prominently.
- These features likely add nuance to individual predictions rather than global shifts in classification.

## Least Important Features:

- Sepsis, Fever, Reusable scope, and Postoperative stent have very minimal influence, with very low importance values.
- These features can be potential candidates for removal in feature selection without compromising model performance significantly.

1. **SHAP Waterfall Plot**


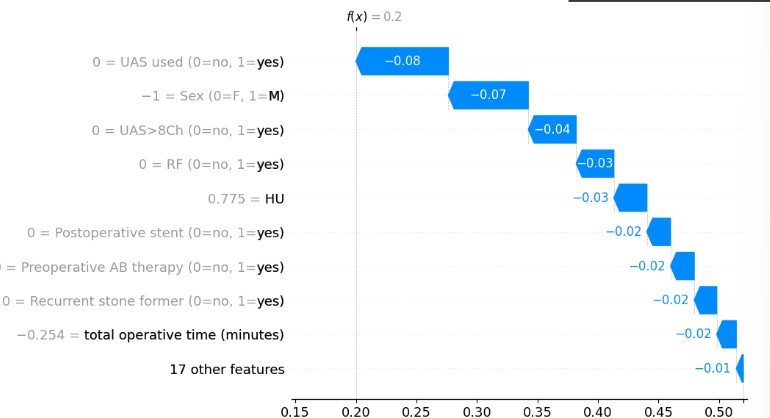


## Purpose: Breaks down a single prediction to show how each feature increased or decreased the model output from the base value.

## Axes & Elements:

- - X-Axis: Model output value (range: 0.20 to 0.50)
    - Left (blue): Lower probability of anomaly
    - Right (pink): Higher probability of anomaly
  - Y-Axis: Features contributing to the prediction
  - Gray line: Baseline prediction (average for all patients)
  - Blue bars: Features that pull the prediction down (toward *no anomaly*)
  - Red bars: Would indicate push toward *anomaly* (none shown here — all push down)

## Key Interpretation:

- - Final predicted probability = 0.20
  - The base value (initial model output before features) is ~0.50
  - Features like:
    - UAS used = 0 → contributed -0.08
    - Sex = Female (0) → contributed -0.07
    - UAS > 8Ch = 0, RF = 0, HU = 971 → further pulled prediction down

## Interpretation: The combination of no UAS usage, female sex, no RF, and moderate HU led to a significantly lower anomaly risk prediction for this patient.

1. **SHAP Force Plot (Prediction-Level Impact Visualization)**


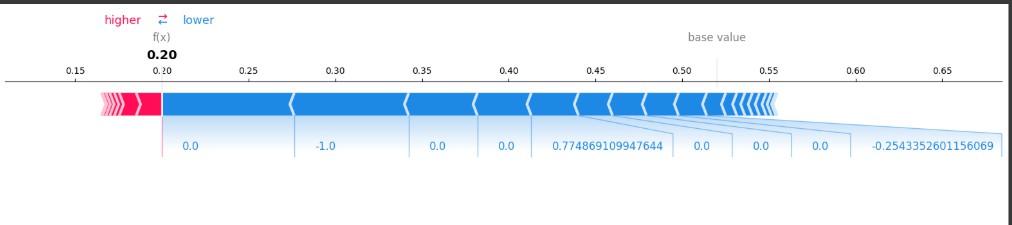


Purpose: Shows how each feature value pushed the prediction up or down for one specific patient.

## Axes & Elements:

- - Center Value (f(x)) = 0.20: Final model prediction for the individual
  - Base Value (typically near 0.50): Average prediction across population
  - Blue Arrows: Features that push prediction lower
  - Red Arrows: Would push prediction higher (none in this example)

## Key Interpretation:

- - Features like:

## UAS used = 0, Sex = Female (0), RF = 0, UAS > 8Ch = 0

- - - All pull the prediction significantly downward
  - The strongest negative contributors are UAS usage and sex
  - Minimal to no features pulling the prediction up → supports low anomaly likelihood

## SHAP Decision Plot (Cumulative Prediction Path)

**
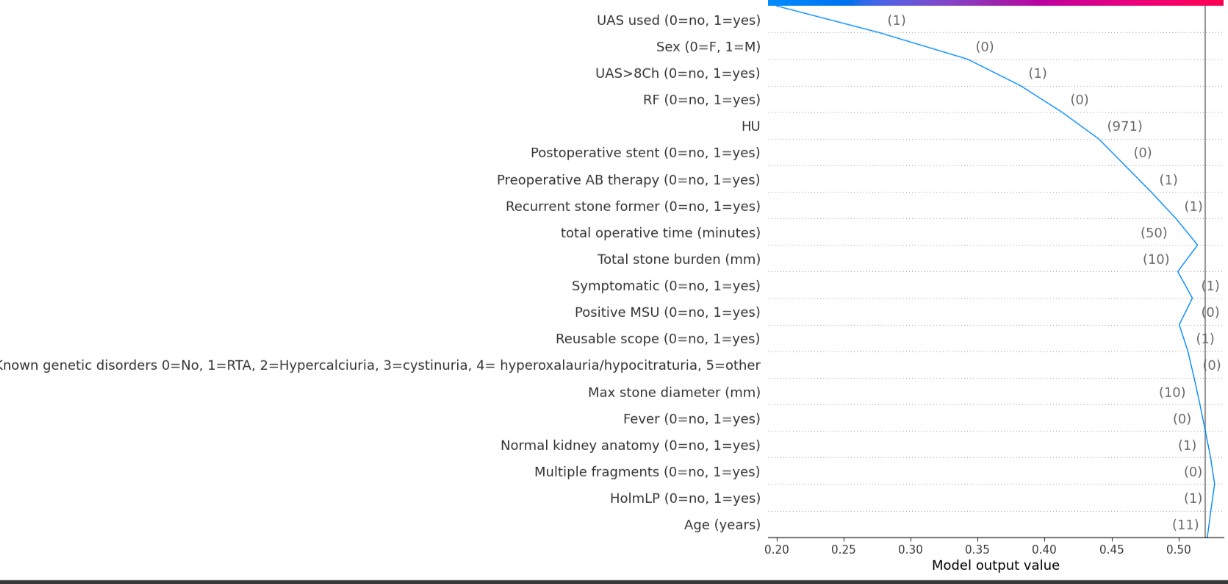
**

Visualizes the cumulative effect of all feature contributions for a single prediction, tracing how the model arrived at the output.

##

## Axes & Elements:

- - X-Axis: SHAP value cumulative contribution to the model output
  - Left (0.15) → pushes toward *no anomaly*
  - Right (>0.50) → pushes toward *anomaly*
  - Horizontal lines: Show feature values and how much each added/subtracted from the base prediction
  - f(x) = 0.20: Final model output

## Key Interpretation:

- - The model started at a **base value ~0.50**
  - Major features like: Sex = Female, UAS used = 0, RF = 0, UAS > 8Ch = 0, and short operative time
  - Each step pushed the prediction lower, reaching a final output of 0.20
  - Highlights a linear additive path, illustrating model interpretability and transparency

## SHAP Summary Plot

**
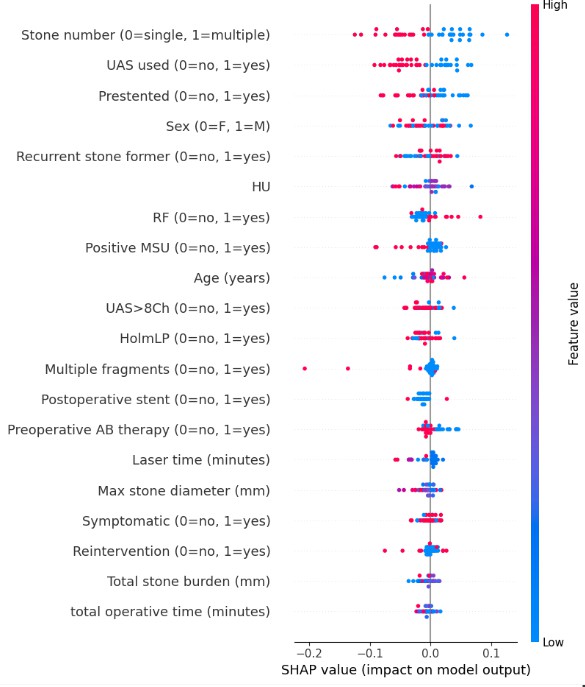
**

**Purpose**: This plot illustrates how each feature contributes to the model’s predictions across all patients, showing both magnitude and direction of impact.

## Axes & Color Coding:

- - **Y-Axis**: Features ranked by **average absolute SHAP value** (i.e., overall importance).
  - **X-Axis**: SHAP value → measures **impact on model output**.
    - **Right (positive)**: pushes prediction toward *“Anomalies”*.
    - **Left (negative)**: pushes prediction toward *“No Anomalies”*.

## Color:

- - - **Red**: High feature value
    - **Blue**: Low feature value

# Top Features (High Predictive Impact):

## Stone number (0=single, 1=multiple)

- - - High values (multiple stones, red) increase the predicted risk of anomalies (positive SHAP).
    - Consistently pulls predictions to the right → strong anomaly indicator.

## Laser time (minutes)

- - - Low laser time (blue): pushes prediction toward *anomalies*.
    - High laser time (red): often linked with *non-anomalous* outcomes — potentially due to more thorough procedures.

## Total stone burden (mm)

- - - Higher burden (red): surprisingly pushes toward *no anomalies*, possibly due to proactive surgical intervention.
    - Indicates a non-linear or compensatory relationship.

## RF (0=no, 1=yes)

- - - RF = 1 (red): tends to increase anomaly risk.

## Presented (0=no, 1=yes)

- - - Patients who presented with symptoms (1) are more likely to show *anomalies*.

# Moderately Important Features:

## UAS used (0=no, 1=yes)

- - - Mixed impact — value-dependent but moderately influential.

## HU (Hounsfield Unit)

- - - Varies across patients; no strong directional pattern, but consistent moderate impact.

## Age (years)

- - - Older patients (red) show some trend toward *higher anomaly prediction*, but less consistent.

## Total operative time (minutes)

- - - Longer durations (red) often push toward *no anomalies* → may indicate careful surgical handling.

## Max stone diameter (mm)

- - - Mixed influence, generally modest.

# Least Important Features:

## Fever, Sepsis, Known genetic disorders, Normal kidney anatomy

- - - SHAP values cluster around zero.
    - These features have **minimal impact** on model decisions — suggesting **low model reliance** on them in the current dataset.
